# Proteomic Study of the Survival and Resuscitation Mechanisms of Filamentous Persisters in an Evolved Escherichia coli Population from Cyclic Ampicillin Treatment

**DOI:** 10.1128/mSystems.00462-20

**Published:** 2020-07-28

**Authors:** Jordy Evan Sulaiman, Henry Lam

**Affiliations:** a Department of Chemical and Biological Engineering, The Hong Kong University of Science & Technology, Kowloon, Hong Kong; Chan Zuckerberg Biohub

**Keywords:** antibiotic, ampicillin, evolution, persisters, resuscitation, filaments, proteomics

## Abstract

Through adaptive laboratory evolution (ALE) experiments, it was recently found that when a bacterial population was repetitively treated with antibiotics, they will adapt to the treatment conditions and become tolerant to the drug. In this study, we utilized an ampicillin-tolerant Escherichia coli population isolated from an ALE experiment to study the mechanisms of persistence during ampicillin treatment and resuscitation. Interestingly, the persisters of this population exhibit filamentous morphology upon ampicillin treatment, and the filaments are getting longer over time. Proteomics analysis showed that proteins involved in carbohydrate metabolism are upregulated during antibiotic treatment, in addition to those involved in the oxidative stress response. Bacterial SOS response, which is associated with filamentation, was found to be induced on account of the increasing expression of RecA. Measurement of endogenous reactive oxygen species (ROS) revealed that the population have ∼100-fold less ROS generation under ampicillin treatment than the wild type, leading to a lower mutagenesis rate. Single-cell observations through time-lapse microscopy show that resuscitation of the filaments is stochastic. During resuscitation, proteins involved in the tricarboxylic acid (TCA) cycle, glyoxylate cycle and glycolytic processes, and ATP generation are downregulated, while ribosomal proteins and porins are upregulated in the filaments. One particular protein, ElaB, was upregulated by over 7-fold in the filaments after 3 h of resuspension in fresh medium, but its expression went down after the filaments divided. Knockout of *elaB* increased persistence on wild-type E. coli, and upon resumption of growth, mutants lacking *elaB* have a higher fraction of small colony variants (SCVs) than the wild type.

**IMPORTANCE** Persisters are a subpopulation of cells with enhanced survival toward antibiotic treatment and have the ability to resume normal growth when the antibiotic stress is lifted. Although proteomics is the most suitable tool to study them from a system-level perspective, the number of persisters that present naturally is too few for proteomics analysis, and thus the complex mechanisms through which they are able to survive antibiotic stresses and resuscitate in fresh medium remain poorly understood. To overcome that challenge, we studied an evolved Escherichia coli population with elevated persister fraction under ampicillin treatment and obtained its proteome profiles during antibiotic treatment and resuscitation. We discovered that during treatment with ampicillin, this tolerant population employs an active oxidative stress response and exhibits lower ROS levels than the wild type. Moreover, an inner membrane protein which has implications in various stress responses, ElaB, was found to be highly upregulated in the persisters during resuscitation, and its knockout caused increased formation of small colony variants after ampicillin treatment, suggesting that ElaB is important for persisters to resume normal growth.

## Abstract (precis)

Persisters are a subpopulation of cells with enhanced survival toward antibiotic treatment and have the ability to resume normal growth when the antibiotic stress is lifted. Although proteomics is the most suitable tool to study them from a system-level perspective, the number of persisters that present naturally is too few for proteomics analysis, and thus the complex mechanisms through which they are able to survive antibiotic stresses and resuscitate in fresh medium remain poorly understood. To overcome that challenge, we studied an evolved Escherichia coli population with elevated persister fraction under ampicillin treatment and obtained its proteome profiles during antibiotic treatment and resuscitation. We discovered that during treatment with ampicillin, this tolerant population employs an active oxidative stress response and exhibits lower ROS levels than the wild type. Moreover, an inner membrane protein which has implications in various stress responses, ElaB, was found to be highly upregulated in the persisters during resuscitation, and its knockout caused increased formation of small colony variants after ampicillin treatment, suggesting that ElaB is important for persisters to resume normal growth.

## INTRODUCTION

Persisters are a subpopulation of cells that have enhanced abilities to survive antibiotics and other stressful conditions. These specific bacterial populations position themselves in a favorable phenotypic niche with abundances of DNA, RNA, proteins, or other cellular components that increase their likelihood of survival (1–4). When the stresses are removed, persisters can resuscitate, giving rise to a new progeny which is identical to the susceptible original population and causes the relapse of infectious diseases (5, 6). Despite the clear clinical importance, their inherent transience, low abundance, and cellular heterogeneity make it challenging to study them.

Recently, several groups have applied cyclic antibiotic treatment to a bacterial population, a form of adaptive laboratory evolution (ALE) experiments, to generate evolved populations with elevated persister fractions. An evident advantage of this method compared to other methods such as chemical pretreatments, toxin overexpression, or nutrient shifts is that it mimics the natural development of persistence in a bacterial population, such as when the bacteria are exposed to antibiotics repeatedly in clinical patients. Fridman et al. exposed Escherichia coli populations to high ampicillin concentrations intermittently with different exposure times and observed that their evolved strains developed tolerance by optimizing the lag time to match the duration of the antibiotic exposure. They traced this adaptation to specific genetic mutations, where the reversion of the wild-type (WT) alleles could restore the antibiotic sensitivity of the original population (7). Van den Bergh et al. also studied the development of elevated persistence in E. coli under repeated antibiotic treatment during the stationary phase and reported that their evolved populations have an ∼1,000-fold increase in the persister cell formation during early stationary phase (8). A newer ALE experiment by Khare and Tavazoie (9) used two antibiotics with orthogonal modes of action (ampicillin and ciprofloxacin, or ampicillin and kanamycin) on exponential-phase E. coli. The selection cycles to generate the tolerant population are much longer than those in the studies by Fridman et al. and Van den Bergh et al., probably due to the drug combination treatment. They found that the mutations driving the persistence phenotype are enriched in translation-related genes (9). Worryingly, a follow-up study by the Balaban group demonstrated that development of resistance is quicker in these tolerant populations from the ALE experiment than in the wild-type population (10). In other words, once the population has attained the mutations that govern tolerance, the chances for new resistance-associated mutations to emerge are amplified. Therefore, studying the mechanisms of these tolerant populations and developing treatment strategies to combat the tolerant cells before they acquire resistance will be of great clinical interest.

Our group has recently performed similar ALE experiments as a means to generate evolved populations with high fractions of persister cells and then subject them to proteomics study for cross-comparison analysis of the regulated proteomes (11). Using this approach, we have identified a set of protein candidates with similar expression profiles across multiple tolerant populations that are important for their persistence phenotype. In this study, we characterize one of the evolved populations generated from the evolution experiment using ampicillin, Evo3A, that have a high fraction of filamentous persisters. By whole-genome sequencing, we detected several point mutations in Evo3A (compared to wild-type E. coli K-12 MG1655): two nonsynonymous single point mutations on *yhgE* (changing the start codon to leucine) and *cyaA* (changing tryptophan to a stop codon in the middle of the gene) genes, and one synonymous mutation on the *ybbA* gene. Evo3A was subsequently found to filament extensively in prolonged ampicillin treatment, and the filamentous cells from Evo3A possess the key characteristics of persisters, namely, the ability to survive a longer-term antibiotic treatment and to resume growth after treatment. We surmise that filamentation is one of the survival strategies of the cells to minimize the need for cell wall synthesis, which is targeted by ampicillin (12). Because of its elevated persister fraction, we were able to collect enough cells to conduct a time course proteomic analysis during antibiotic treatment and resuscitation, which was not possible with wild-type E. coli. Besides, the unique filamentous morphology of the persisters also serves as a marker to conveniently track their resuscitation and enable their isolation from new progenies which already undergo cell division. By comparing the proteomes of the filaments and the new progenies, we were able to reveal the protein markers that appear to play in role in the resuscitation process.

## RESULTS

### Persisters from Evo3A show extensive filamentation during ampicillin treatment.

**FIG 1:**
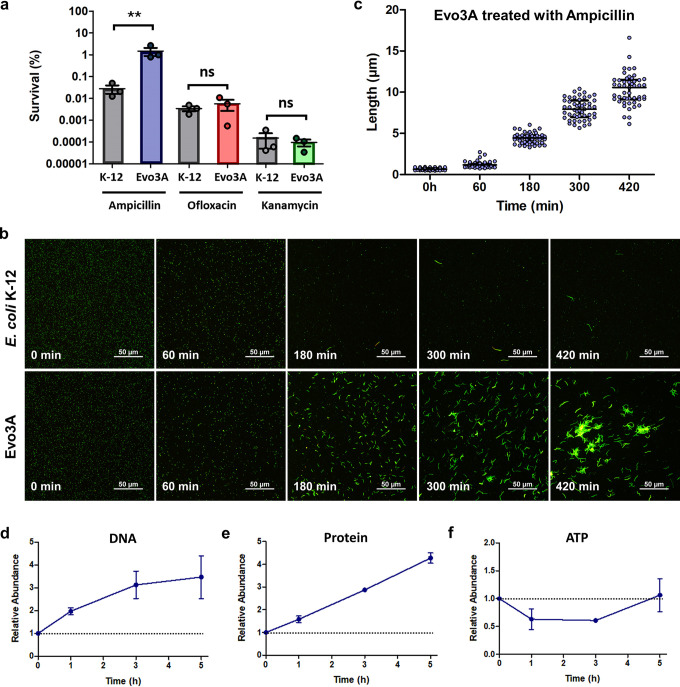
Evo3A has increased survival toward ampicillin treatment, and the persisters exhibit extensive filamentation. (a) Survival (%) of E. coli K-12 and Evo3A to ampicillin (125 μg/ml), ofloxacin (5 μg/ml), and kanamycin (150 μg/ml) treatment for 3 h (mean ± standard error of the mean [SEM], *n* = 3). Significance of difference with ancestral population: **, *P* < 0.01; ns, not significant (two-tailed *t* test with unequal variances of the log-transformed values). (b) Epifluorescence microscopy of E. coli K-12 and Evo3A during antibiotic treatment. Cells before and after 1, 3, 5, and 7 h of ampicillin treatment (∼15× MIC) were stained with a LIVE/DEAD kit and visualized by epifluorescence microscopy. Green cells are viable cells, and red cells are dead cells. (c) Length of ampicillin-treated Evo3A cells, calculated by averaging 50 distinct individual cells randomly from each time point (data were shown as median with interquartile range, *n* = 50). (d to f) DNA (d), protein (e), and ATP (f) extracted from Evo3A before and after 1, 3, and 5 h of ampicillin treatment. DNA was extracted using Qiagen’s DNeasy blood and tissue kit, protein concentrations were determined by the bicinchoninic acid (BCA) assay, and ATP measurement was performed using the BacTiter-Glo assay. Data are presented relative to the untreated cells (0 h), as marked by the horizontal dotted lines. Data represent three biological replicates, and each data point is denoted as mean ± SEM.

Evo3A is an evolved E. coli population from a recent adaptive laboratory evolution (ALE) experiment (11), possessing nonsynonymous single point mutations on *yhgE* and *cyaA* genes that likely render them nonfunctional. To generate Evo3A, E. coli K-12 MG1655 was treated with a high dose of ampicillin (∼10× MIC) during exponential phase, the survivors were regrown on fresh medium, and the cycle was repeated for four times. When Evo3A was exposed to a high concentration of ampicillin, we observed that it had over a 10-fold increase in survival compared to the wild-type strain (Fig. 1a), without an increase in MIC (8 μg/ml). However, when treated with other classes of antibiotics, the evolved population showed no increase in survival over the wild-type strain, hence showing no cross-tolerance to other types of drug. Similarly, it has been reported that a Δ*cyaA* mutant has an increased survival compared to the WT upon exposure to cell wall-acting antibiotics, including ampicillin, but not to other antibiotics, like ciprofloxacin or gentamicin (13). As Evo3A highly resembles the Δ*cyaA* mutant, where the single point mutation on Evo3A’s *cyaA* gene led to an amino acid change to a stop codon at the middle of the gene, our observation is consistent with the previously reported result. As shown previously, Evo3A has a high persistence phenotype, where the mutations confer tolerance to about 1% of the population, resulting in a bimodal killing curve (11).

We observed both E. coli K-12 and Evo3A under the microscope before and after treatment with lethal doses of ampicillin (∼15× MIC) (Fig. 1b). While most E. coli K-12 cells died, with a few persisters surviving the treatment, Evo3A showed high survivability upon ampicillin treatment, and the surviving cells had filamentous morphology, which is an indication of an induced SOS response (14). Interestingly, when subjected to prolonged antibiotic treatment, the filaments are getting longer over time, unlike E. coli K-12, where the filaments stop growing after treatment for ∼3 h (Fig. 1b and c). After 7 h of ampicillin treatment, we observed that the filaments on Evo3A started to form clusters due to the increased length of the filaments.

Although cell division is blocked during filamentation, we wanted to know whether cellular growth and DNA replication still continue in Evo3A. By extracting the total DNA from Evo3A before and after ampicillin treatment, we confirmed that the cells indeed have an increasing DNA amount during the longer treatment durations, suggesting that the filaments may be akin to multiple daughter “cells” strung together without dividing (15) (Fig. 1d). We also measured the protein concentration and ATP level at different time points during antibiotic treatment, which serve as important growth-related cellular components (Fig. 1e and f), and observed that the content of protein and ATP are increasing over the duration of antibiotic treatment on Evo3A. The decrease in ATP level from 0 to 1 h after treatment is attributable to the sharp decrease in cell number during the first hour of treatment (first phase of killing of the biphasic curve [11]). Taken together, we showed that during antibiotic treatment, the surviving filaments are loaded with machinery necessary for growth but do not undergo cell division, perhaps to evade the effect of ampicillin, which targets the cell wall synthesis machinery.

### Resuscitation of the filaments is stochastic, resembling the persisters in wild-type E. coli.

**FIG 2:**
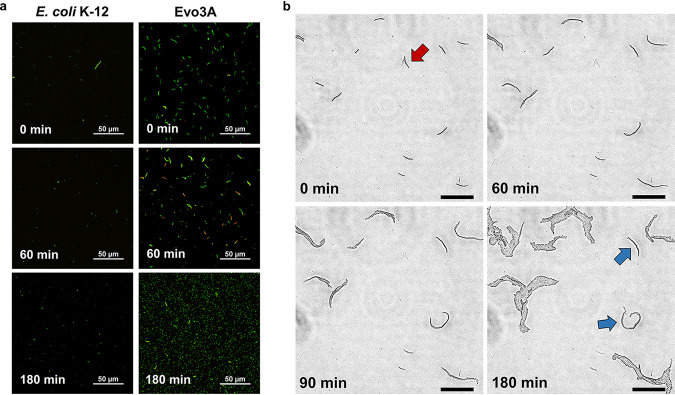
Microscopy of persisters from E. coli K-12 and Evo3A during resuscitation after ampicillin treatment. (a) Cells exposed to ampicillin (∼15× MIC) for 5 h were resuspended in fresh LB medium and observed by epifluorescence microscopy at indicated time points during resuscitation. Green cells are live cells while red cells are dead cells (defined as those with compromised membrane). (b) Time-lapse microscopy of Evo3A during resuscitation on LB pads. Figures are representative phase-contrast images of recovering ampicillin-treated Evo3A cells on LB agar pads, and the panel at *t* = 180 min shows the incipient colony formation from a filament. Red arrow shows a lysed filament, and blue arrows indicate cell division events that are slower than the other cells. Bars, 50 μm.

We realize that the filamentous morphology of the persisters on Evo3A gives us a convenient way to study resuscitation, as the cell length serves as a visual marker for resuscitation upon resuspension in fresh medium. To track the population-wise resuscitation event, we observed both E. coli K-12 and Evo3A under the microscope upon growth in fresh LB medium after 5 h of ampicillin treatment (Fig. 2a). From the microscope images, the persisters on Evo3A appeared to divide faster than those from the WT. For both E. coli K-12 and Evo3A, some filaments already divide into new normal-size cells after 1 h, but the cell division mainly happens between 1 and 3 h upon resuspension in fresh medium. After 1 h, we could clearly see that some of the filaments turned red (dead) and some were still green (viable) on Evo3A. We also spotted the presence of normal-size cells among the filaments. This suggests that the first hour is important for determining whether the filaments will divide or lyse. Also, the resuscitation of the filaments seems to be asynchronous and stochastic, resembling the resuscitation of persister cells in WT E. coli, which have high cell-to-cell variability in awakening times (16). After 3 h of growth, we could still see some viable filaments that had not divided, although the population now consisted of mainly new progenies. To observe resuscitation at a single-cell level, we performed time-lapse microscopy on Evo3A’s filaments upon resuscitation on LB pads, and similar phenomena were observed (Fig. 2b). We noticed that some filaments were lysed (red arrow) during the first hour, and some filaments underwent a slower division process than the others (blue arrow), indicating the stochasticity of the resuscitation event. Again, we saw that the cell division mainly happened sometime between 1 and 3 h upon growth on LB pads.

### Time-series proteomics analysis of Evo3A during ampicillin treatment.

**FIG 3:**
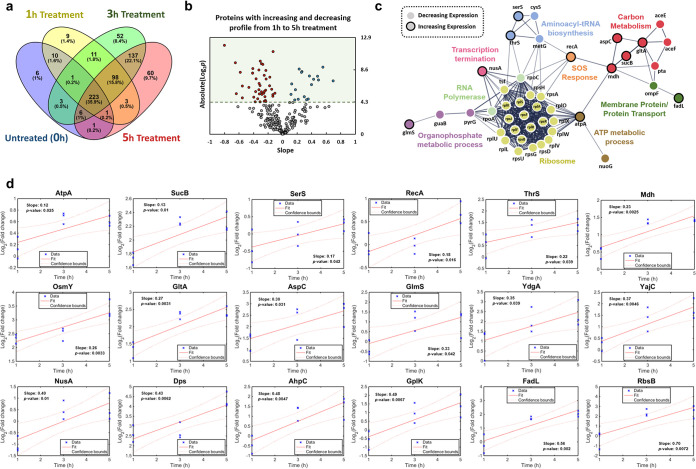
Time-series analysis of the protein expression profile on Evo3A during antibiotic treatment. (a) Venn diagram for proteome comparison of Evo3A before and after 1 h, 3 h, and 5 h of ampicillin treatment (125 μg/ml). (b) Volcano plots of the protein expression profile on Evo3A from 1 h to 5 h of ampicillin treatment. Fold change values of the proteins after 1 h, 3 h, and 5 h of treatment are fitted to a linear regression model, and the slopes of the fitted curve were shown with the respective *P* value for each protein. Slopes with a *P* value below 0.05 or an absolute log_2_(*P* value) above 4.32 are considered significant. Proteins with increasing expression profiles are marked by the blue dots, while proteins with decreasing expression profiles are marked by the red dots. (c) Protein-protein interaction network of the proteins with increasing and decreasing expression profiles throughout the antibiotic treatment as predicted by STRING v10.5. The lines represent protein interaction (thicker lines mean higher confidence), and the dots in different colors represent different biological processes involved. Nodes with black outlines are proteins with increasing expression profile, and nodes with no outlines are proteins with decreasing expression profile. Nodes without function enrichment are hidden from the network. (d) Linear model fitting of the 18 proteins with increasing expression profile during antibiotic treatment from 3 biological replicates of Evo3A. Red lines represent the fitted linear models with the 95% confidence bounds shown as red dotted curves.

To observe the proteome changes on Evo3A during antibiotic exposure, we performed shotgun proteomics on Evo3A after 1 h, 3 h, and 5 h of ampicillin treatment. We employed our previously developed magnetic bead-based separation approach to remove the dead cells after antibiotic treatment and enrich for persisters (3). Combining all replicates, 356, 531, and 529 distinct proteins were identified for Evo3A after 1 h, 3 h, and 5 h of ampicillin treatment, respectively, while 251 proteins were identified for Evo3A cells before ampicillin treatment (Fig. 3a). To capture any increasing or decreasing trends of any protein expression profile in Evo3A during the antibiotic treatment, we fit the fold changes (relative to the one before treatment) of proteins commonly observed at all time points (Fig. 3a) to a linear regression model. Figure 3b shows the volcano plot of the slopes of the fitted lines, against the *P* values. Proteins with significant slope (different from zero) were defined as those with *P* values below 0.05. A total of 18 and 42 proteins were found to have increasing and decreasing expression profiles, respectively, during ampicillin treatment (see Table S2 in the supplemental material), and these proteins may be important for the filamentation process. From the protein-protein interaction network (Fig. 3c), we can see that most of the proteins such as ribosomal proteins, RNA polymerases, and some proteins for pyruvate metabolism such as AceE and AceF have a decreasing expression profile on Evo3A during antibiotic treatment. Most proteins for carbohydrate metabolism, however, have increasing expression, such as citrate synthase (GltA), SucB, and malate dehydrogenase (Mdh) in the tricarboxylic acid (TCA) cycle. Interestingly, we found that RecA, one of the key players in the SOS response, has an increasing expression profile during the antibiotic treatment. RecA induces the SOS response following DNA damage (17), and the expression of RecA has been used as an indicator of induced SOS response and DNA damage in E. coli (1, 18). It is known that SOS response leads to cell filamentation (14), like what we observed in Evo3A. Other proteins with increasing expression profiles during antibiotic treatment include OsmY, which is induced by stresses known to cause protein misfolding, such as DNA damage from oxidative stress; DNA starvation protection protein (Dps), which protects DNA from oxidative damage; AhpC, the primary scavenger for endogenously generated hydrogen peroxides for cell protection against oxidative stress; and NusA, which is important for the coordination of cellular responses to DNA damage.

### Persisters from Evo3A employ an active oxidative stress response and generate less reactive oxygen species (ROS) than the WT.

**FIG 4:**
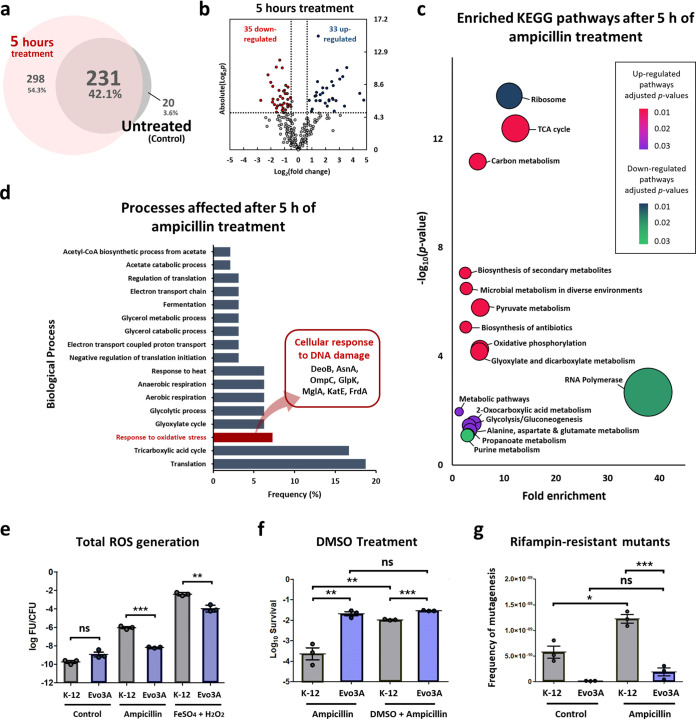
Proteome comparison of Evo3A after 5 h of ampicillin treatment to that before treatment. (a) Venn diagram for proteome comparison of Evo3A before and after 5 h of ampicillin treatment (125 μg/ml). (b) Volcano plot for Evo3A after 5 h of ampicillin treatment compared with Evo3A before ampicillin treatment. Red dots are the downregulated proteins, and blue dots are the upregulated proteins. (c) Pathway enrichment study (KEGG) by DAVID of the differentially expressed proteins and newly detected proteins on Evo3A after 5 h of ampicillin treatment compared to those before treatment. Fold enrichment is defined as the ratio of proportion of the input information to the background information. (d) Functional classification of the differentially expressed and newly detected proteins on Evo3A after 5 h of ampicillin treatment compared to those before treatment by gene ontology (GO) analysis using DAVID, classified by biological process. (e) Total ROS generation in cultures treated with ampicillin (125 μg/ml) or 50 μM FeSO_4_ and 10 mM H_2_O_2_ for 1 h was assayed using H_2_DCFDA probe and normalized by CFU counts (mean ± SEM, *n* = 3). (f) Survival of cultures exposed to ampicillin (125 μg/ml) for 5 h in the presence or absence of 5% DMSO (an OH^·^ scavenger). Aliquots were washed and plated to determine survival (mean ± SEM, *n* = 3). Significance of difference: ***, *P* < 0.001; **, *P* < 0.01; ns, not significant (two-tailed *t* test with unequal variances of the log-transformed values). (g) Frequency of mutagenesis was assayed in cells previously exposed to ampicillin for 1 h. Cultures were washed, grown overnight, and plated on LB agar plates supplemented with rifampin (100 μg/ml). Changes in the frequency of generation of rifampin-resistant mutants were used as an indicator for DNA damage (mean ± SEM, *n* = 3). Significance of difference: ***, *P* < 0.001; *, *P* < 0.05; ns, not significant (two-tailed *t* test with unequal variances).

To see the proteome changes on Evo3A after prolonged antibiotic treatment, we directly compared the proteome profile of Evo3A after 5 h of ampicillin treatment to those before treatment (Fig. 4a). Figure 4b shows the volcano plot of fold changes (relative to the untreated control) against *P* values obtained from the *t* test. A total of 68 proteins (35 downregulated and 33 upregulated) were differentially expressed in the 5-h-ampicillin-treated Evo3A compared to the untreated culture. The list of differentially expressed proteins is shown in Table S3. Because proteins that were identified during 5 h of antibiotic treatment but not identified before the treatment should also be upregulated and potentially important, we further filtered those proteins with high numbers of peptide spectrum matches (>5) and refer to them as the newly detected proteins (Table S4), to be combined with the differentially expressed proteins for pathway enrichment and gene ontology (GO) analysis. The enriched pathways (KEGG) predicted by DAVID (19) given the set of differentially expressed and newly detected proteins after 5 h of treatment are shown in Fig. 4c. We observed an upregulation in several pathways such as TCA cycle, carbon metabolism, and pyruvate metabolism, which are common bacterial responses against antibiotics (20, 21), and also pathways that modulate the oxidative stress response, such as glutamate metabolism (22, 23), glyoxylate and dicarboxylate metabolism (glyoxylate cycle) (24), and propanoate metabolism (25). In addition, ribosomal proteins, RNA polymerases, and proteins for purine metabolism were downregulated after 5 h of treatment.

We used gene ontology (GO) analysis by DAVID (19) to categorize the differentially expressed and newly detected proteins identified in Evo3A after 5 h of ampicillin treatment and determined several pathways that were known to play a role in persister formation, especially the oxidative stress response (Fig. 4d). To evaluate the oxidative stress in the cells, we first measured the endogenous ROS production in Evo3A and WT E. coli after exposure to ampicillin by using the fluorescent probe 2′,7′-dichlorodihydrofluorescein diacetate (H_2_DCFDA) (Fig. 4e). We observed ∼100-fold less ROS generation in the ampicillin-treated Evo3A than in the ampicillin-treated WT. As a positive control, we also found that Evo3A also exhibited lower ROS generation in Evo3A than the WT when treated with the Fenton reagents (FeSO_4_ and H_2_O_2_) that generate hydroxyl radicals (26). This observation is in line with the activated oxidative stress response observed from our proteomics data. Together, these explain the microscopic observation in Fig. 1b that while the filaments from WT E. coli stop becoming longer at ∼3 h after antibiotic treatment and probably lyse due to the excessive accumulation of ROS, the filaments from Evo3A are less prone to lysis due to the lower accumulated ROS. Moreover, we also observed an ∼10-fold increase in the survival of WT E. coli when exposed to ampicillin in the presence of dimethyl sulfoxide (DMSO), which is an OH^·^ scavenger (27), but no difference in the survival of Evo3A (Fig. 4f). This suggests that OH^·^ is a good indicator for the ROS produced by cells during ampicillin treatment, as DMSO is able to greatly increase the survival of WT. Moreover, one can also conclude that there should be less OH^·^ in Evo3A under ampicillin treatment than in the WT, since DMSO has no effect, further confirming our observation in the ROS assay.

As oxidative stress due to hydroxyl radical generation usually causes DNA damage, we wanted to see the degree of DNA damage caused by ampicillin treatment to the cells. We evaluated the mutagenesis rate of Evo3A and the WT strain by observing the changes in the frequency of generation of rifampin-resistant mutants after treatment with ampicillin. From Fig. 4g, we can see that the wild-type E. coli shows a higher mutagenesis rate when treated with ampicillin than the untreated one, as demonstrated previously (28, 29), while Evo3A has negligible change in the mutagenesis rate under ampicillin treatment compared to the untreated one. In addition, the mutagenesis rate in Evo3A is much lower than that of the WT under ampicillin treatment, which is likely due to a much reduced OH^·^ level after antibiotic treatment compared to the WT. This is also consistent with the observation that Evo3A shows no difference in survival upon treatment with ampicillin in the presence or absence of DMSO, the OH^·^ scavenger (Fig. 4f).

We also observed the upregulation of several proteins that act as cellular responses to DNA damage such as DeoB, AsnA, OmpC, GlpK, MglA, KatE, and FrdA (Fig. 4d), further confirming that ampicillin treatment causes DNA damage on the cells. Moreover, among the differentially expressed proteins after 5 h of antibiotic treatment are those involved in the regulation of translation and protein folding. This might be due to an increase of protein aggregation arising from the inactivation of DnaK chaperone after exposure to oxidative stress (30). Indeed, from our proteomics data, we observed that DnaK chaperone was downregulated by 1.44-fold (*P* value 0.019) but is not considered to be differentially expressed in our result because its downregulation is below 1.5-fold. Several ATP synthase proteins such as AtpA, AtpD, and AtpG are upregulated after antibiotic treatment (Tables S2 and S3), confirming that the cells have increased ATP levels during treatment (Fig. 1f).

### Enrichment and proteomics analysis on the filamentous persisters during resuscitation.

Due to the higher number of persisters on Evo3A than on WT, we were also able to perform shotgun proteomics on the surviving filaments during resuscitation (at 1, 2, and 3 h after resuspension in fresh medium) (Fig. S1). The list of differentially expressed proteins from sample after 1 h, 2 h, and 3 h of resuspension in fresh medium compared to those before resuspension is shown in Table S5. From the protein-protein interaction network (Fig. S1g to i), we could see that as opposed to those during antibiotic treatment, the cells upregulate ribosomal proteins while downregulating proteins that are involved in the metabolic processes such as TCA and glyoxylate cycle.

10.1128/mSystems.00462-20.1 FIG S1 Proteomics analysis of Evo3A after resuspension in fresh medium. Download FIG S1, TIF file, 1.2 MB. Copyright © 2020 Sulaiman and Lam. 2020 Sulaiman and Lam This content is distributed under the terms of the Creative Commons Attribution 4.0 International license.

**FIG 5:**
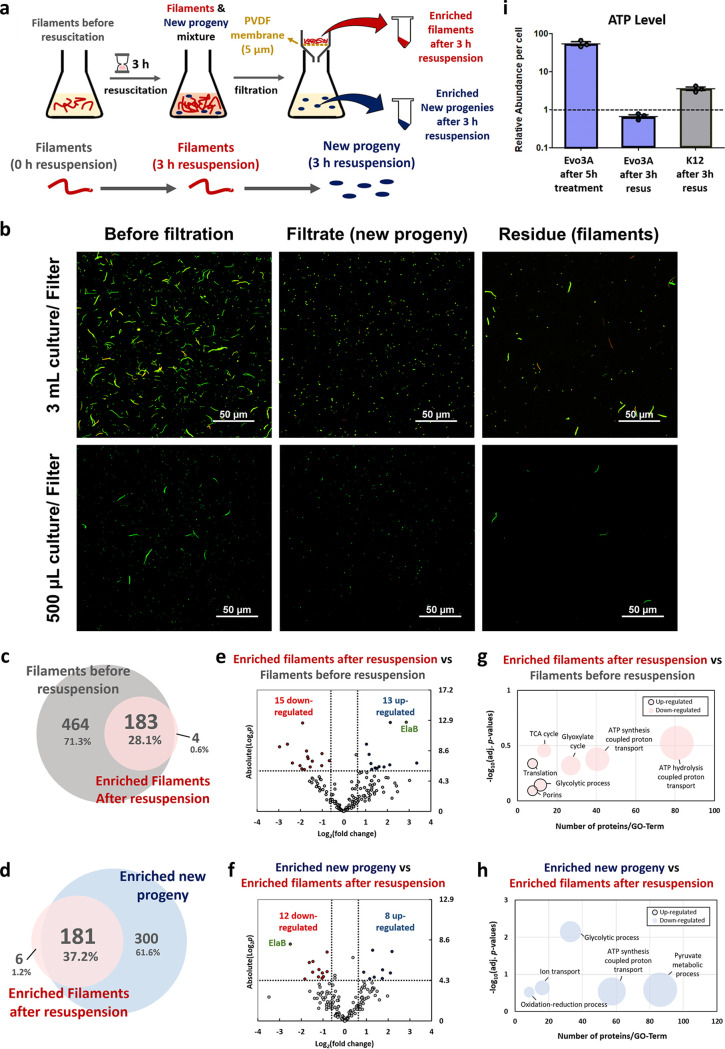
Separation of filaments from the new progeny after 3 h of resuspension in fresh medium. (a) Workflow for separation of filaments from the new progeny. A mid-exponential culture of Evo3A was treated with ampicillin (∼15× MIC) for 5 h, washed to remove antibiotic, and resuspended in fresh LB medium. After 3 h of resuspension, there was a mixture of filaments and newly divided cells. The mixture was filtered with a PVDF membrane (5-μm pore size, 25-mm diameter) to separate the filaments (residue) and the normal-sized new progeny (filtrate). (b) Epifluorescence microscopy of the cells before and after filtration. The top row shows filtration using one filter for 3 ml culture, and the bottom row shows filtration using 6 filters for 3 ml culture (one filter per 500 μl). (c and d) Venn diagrams for proteome comparison of the enriched filaments to those before resuspension (c) and the enriched new progeny to the enriched filaments (d). (e and f) Volcano plots of the enriched filaments compared to those before resuspension (e) and the enriched new progeny compared to the enriched filaments (f). Differentially expressed proteins are defined as those with Benjamini-Hochberg-corrected *P* values below 0.1, and fold change higher or lower than ±1.5, corresponding to the colored dots. Red dots are the downregulated proteins, and blue dots are the upregulated proteins. (g and h) Functional classification of the differentially expressed proteins identified in the enriched filaments compared to those before resuspension (g) and the enriched new progeny compared to the enriched filaments (h) by gene ontology (GO) analysis using DAVID, classified by biological process. Dot size is proportional to the *y* axis. (i) ATP level of Evo3A after 5 h of ampicillin treatment, Evo3A after 3 h of resuscitation, and WT after 3 h of resuscitation, compared to those before treatment, measured using the BacTiter-Glo assay. To calculate the abundance on a per-cell basis, the measured values were normalized by CFU counts by serially diluting and plating washed aliquots. Data are presented relative to the untreated cells (marked by the horizontal dashed line). Data represent three biological replicates, and each data point is denoted as mean ± SEM.

Since we observed that cell division happens sometime between 1 and 3 h after resuspension in fresh medium (Fig. 2a and b), the proteome profile in Fig. S1 is a mixture between the filaments and the new progeny, especially those after 3 h of regrowth in fresh medium. To isolate the filaments from the new progeny, a polyvinylidene difluoride (PVDF) membrane with a 5-μm pore size is used (Fig. 5a). As seen from Fig. 5b, the filtrate is filament free, but the retentate still contains some normal-size cells when 3 ml culture is filtered with a single filter. We suspect that the filter’s capacity is overwhelmed with excessive cell concentrations, and the normal-sized cells are stuck in the filter due to the aggregated filaments. By filtering a smaller amount of cell culture per filter, we show that we could specifically separate the filaments from the new progeny (Fig. 5b). We then compare the enriched filaments after 3 h of resuspension in fresh medium and the enriched new progeny at that time point by quantitative proteomics.

We consider the enriched filaments (after 3 h of resuspension in fresh medium) an “intermediate state” of the persisters, which have the potential to divide into new progenies but have yet to do so (Fig. 5a). It is therefore interesting to observe the proteome changes that accompany the transition from the filaments to the new progenies, to reveal potential protein markers that govern this resuscitation event. Figure 5c to h show the proteome profile of the enriched filaments and the enriched new progeny. Combining all replicates, 187 and 481 distinct proteins were identified in the enriched filaments and enriched new progeny, respectively, while 647 proteins were identified in the filaments before resuspension (Fig. 5c and d). Figure 5e and f show the volcano plots of fold changes against *P* values obtained from the *t* test. The list of differentially expressed proteins from the enriched filaments compared to the one before resuspension is shown in Table S6. The bubble plot in Fig. 5g shows that after 3 h of resuspension, the enriched filaments downregulate proteins for carbon metabolism (TCA and glyoxylate cycle) while they upregulate proteins that play a role in the translation process, consistent with the results obtained in Fig. S1. Besides, the enriched filaments also upregulate proteins involved in protein transport and porins (OmpC, OmpF, and OmpN) that act as pores for passive diffusion. One of the most upregulated proteins in the enriched filaments is ElaB, an inner membrane protein that has been previously implicated in E. coli persistence (31). In addition, after 3 h of resuspension, the filaments have decreased expression of ATP synthase proteins (AtpA and AtpD) compared to those before resuspension. Interestingly, six out of the 15 downregulated proteins on the enriched filaments (NusA, AtpA, AhpC, YajC, OsmY, and GltA) are actually those with an increasing expression profile during the 5 h of antibiotic treatment (Fig. 3d). In contrast, 10 out of the 13 upregulated proteins on the enriched filaments (RlpW, RplX, RplF, RpsF, RpsG, RpoC, Tsf, AceF, OmpF, and OmpC) are those with a decreasing expression profile during the 5 h of antibiotic treatment (Table S2). This means that the expression profile of the filaments during the resuscitation phase is somehow the opposite of that during antibiotic treatment.

Table 1 shows the list of differentially expressed proteins of the enriched new progeny compared to the enriched filaments. From the bubble plot in Fig. 5h, we could see that most of the processes are downregulated during the transition from the filaments to the new progeny. Again, we observed that the new progeny has lower expression of porins (OmpN, OmpA, and OmpC), which are upregulated in the enriched filaments, and also lower expression of ATP synthases. We directly measured the ATP level on Evo3A cells after 3 h of resuspension (Fig. 5i), and we observed that the ATP level on the cells indeed returned to around the same value as that before antibiotic treatment, suggesting that the cells are returning to their “normal state.” This corroborates our proteomic data, which show that the ATP synthase proteins are downregulated on Evo3A during the resuscitation phase. Also, ElaB was observed to be downregulated by 5.5-fold in the new progeny compared to the enriched filaments, indicating that ElaB is overexpressed in the enriched filaments but no longer upregulated once they undergo cell division. This, combined with the fact that ElaB is upregulated in the enriched filaments when cultured in fresh medium, but not during antibiotic treatment, implies that ElaB might be important for the transition of the filamentous persisters to new progenies.

**TABLE 1:** List of differentially expressed proteins of the enriched new progeny (after division) compared to the enriched filaments (before division) after 3 h of resuspension in fresh medium

UniProt code	Gene	Protein name	*P* value	Fold change
P0AEH5	*elaB*	Protein ElaB	0.003	0.18
P08622	*dnaJ*	Chaperone protein DnaJ	0.046	0.28
P77747	*ompN*	Outer membrane porin N	0.014	0.32
P0AC41	*sdhA*	Succinate dehydrogenase flavoprotein subunit	0.028	0.36
P0ABT2	*dps*	DNA protection during starvation protein	0.013	0.36
P0ABA0	*atpF*	ATP synthase subunit b	0.040	0.43
P06959	*aceF*	Dihydrolipoyllysine-residue acetyltransferase component of pyruvate dehydrogenase complex	0.023	0.44
P0AFG8	*aceE*	Pyruvate dehydrogenase E1 component	0.043	0.49
P0A8T7	*rpoC*	DNA-directed RNA polymerase subunit beta′	0.030	0.49
P0A9P0	*lpdA*	Dihydrolipoyl dehydrogenase	0.038	0.51
P0A910	*ompA*	Outer membrane protein A	0.027	0.57
P06996	*ompC*	Outer membrane porin C	0.006	0.57
P0ABB0	*atpA*	ATP synthase subunit alpha	0.028	1.84
P76372	*wzzB*	Chain length determinant protein	0.045	2.23
P00956	*ileS*	Isoleucine-tRNA ligase	0.005	2.45
P10384	*fadL*	Long-chain fatty acid transport protein	0.040	2.56
P0AFF6	*nusA*	Transcription termination/antitermination protein NusA	0.044	3.32
P0A799	*pgk*	Phosphoglycerate kinase	0.023	3.36
P0A917	*ompX*	Outer membrane protein X	0.029	4.33
P0A7U7	*rpsT*	30S ribosomal protein S20	0.006	4.54

### Knockout of *elaB* increases persistence and leads to small colony variant (SCV) formation after ampicillin treatment.

One of the most upregulated proteins in the enriched filaments after 3 h of resuspension in fresh medium is ElaB (above 7-fold increase) (Table S6), a C-tail-anchored inner membrane protein which is implicated in various stress responses. The gene *elaB* is a member of the RpoS regulon, and it is induced during stationary phase or under growth-limited conditions. ElaB localization was checked by tagging ElaB with green fluorescent protein (GFP) at the C terminus using pCA24N-*elaB-gfp* plasmid (Fig. S2a). We observed that the GFP-fused ElaB was localized at the cell poles, suggesting that ElaB is anchored in the membrane, as previously shown by Guo and colleagues (31). However, while normal E. coli cells have only one or two fluorescent dots located at each pole, E. coli cells with filamentous morphology have multiple fluorescent dots located within the cell. This seems to suggest that the E. coli filaments consist of compartments separated by inner membrane-like structures (1, 32), where ElaB proteins are localized.

10.1128/mSystems.00462-20.2 FIG S2 Localization of ElaB protein and knockout of *elaB* gene on E. coli. Download FIG S2, JPG file, 1.0 MB. Copyright © 2020 Sulaiman and Lam. 2020 Sulaiman and Lam This content is distributed under the terms of the Creative Commons Attribution 4.0 International license.

**FIG 6:**
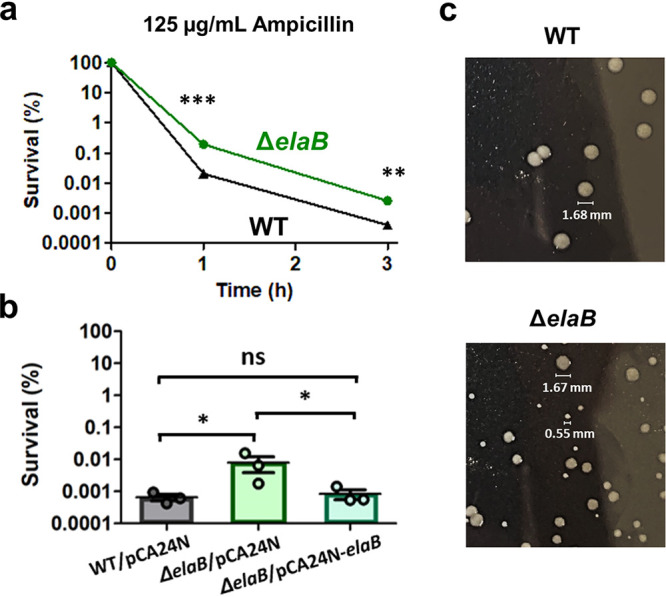
Importance of ElaB in WT E. coli. (a) Survival of wild-type E. coli BW25113 (WT) and Δ*elaB* cells after treatment with ampicillin (125 μg/ml) at the indicated time points (mean ± SEM, *n* = 3). Significance of difference with ancestral population: ***, *P* < 0.001; **, *P* < 0.01 (two-tailed *t* test with unequal variances of the log-transformed values). (b) Survival upon expression of *elaB* via pCA24N-*elaB* in the Δ*elaB* cells was determined with ampicillin (125 μg/ml) treatment for 3 h (mean ± SEM, *n* = 3). *, *P* < 0.05; ns, not significant (two-tailed *t* test with unequal variances of the log-transformed values). Overnight cultures at 1:1,000 were grown in fresh LB until the turbidity at 600 nm reached 0.3, and 1 mM IPTG was added for 2 h. The cells were then treated with ampicillin (125 μg/ml) for 3 h, and survival was measured. Empty plasmid pCA24N was used as a negative control. (c) Colony morphologies for WT and Δ*elaB* cells after 3 h of resuscitation. After treatment with ampicillin (125 μg/ml) for 3 h, cells were washed to remove antibiotics and resuspended in fresh LB for 3 h. After 3 h of growth, aliquots of both WT and Δ*elaB* strains were serially diluted and plated on LB agar (37°C, 20 h of incubation time). Images shown are representative images from 3 biological replicates.

To investigate the importance of ElaB for persistence in WT E. coli, we compared Δ*elaB* mutant and WT E. coli during antibiotic treatment and resuscitation. From Fig. 6a, we could see that the Δ*elaB* mutant has higher survival when treated with ampicillin (125 μg/ml) than does WT after 1 h and 3 h of treatment, indicating that the mutant has higher persistence, as previously reported (31). The number of persister cells formed in the Δ*elaB* strain was around 7-fold higher after 3 h of ampicillin treatment. Additionally, complementation of *elaB* via pCA24N-*elaB* also reduced persistence compared to Δ*elaB* cells harboring an empty pCA24N plasmid (Fig. 6b). If we track the population-wise growth of WT and Δ*elaB* persisters from ampicillin treatment, we see no difference in both growth resumption and growth rate upon inoculation in fresh medium (Fig. S2b). However, when the persisters from the Δ*elaB* mutant are plated on agar medium, we observe the presence of small colony variants (SCVs) during resuscitation (Fig. 6c), which are defined as colonies whose size is around 5 times smaller (or radius 2.23 times smaller) than the most common colony type (33). This SCV formation was not observed on the WT and Δ*elaB* cells when grown under normal conditions without antibiotics, nor on the WT cells that have been treated with a lethal dose of ampicillin (125 μg/ml) for 3 h and resuspended in fresh LB medium (Fig. S2c). This suggested that ElaB should play a role in the resuscitation process of E. coli persisters, as absence of ElaB appears to hamper growth resumption after ampicillin treatment.

## DISCUSSION

In this study, we performed time course proteome profiling on evolved E. coli populations with high persister fractions upon treatment with ampicillin and also during resuscitation. Ampicillin works by inhibiting multiple penicillin binding protein (PBP) targets including PBP3, also known as FtsI, which is a transpeptidase that is essential for peptidoglycan synthesis during cell division (12). Therefore, β-lactam antibiotics that target PBP3, including ampicillin, will interfere with the cell division process and eventually cause cell death. However, binding to PBP3 and blocking its function are only the first step in the action mechanism of ampicillin. As previously described by Dwyer and colleagues, downstream of the target-specific interactions of many antibiotics, including ampicillin, complex redox alterations are triggered which contribute to cellular damage and eventually death (34).

Exposure to β-lactam antibiotics at a certain dose and duration may induce cell filamentation, especially those that primarily target PBP3 such as cephalexin and piperacillin (16, 32). Other groups have also observed the filamentation of E. coli cells when exposed to ampicillin (35, 36), although the fraction of filamentous cells from ampicillin treatment is not as high as we observed in Evo3A. Ampicillin-induced filamentation was consistent with the previously reported ability of this β-lactam to stimulate the SOS response (37, 38). In WT E. coli cells, the majority of the population dies upon ampicillin treatment, leaving a small percentage of filaments which will eventually lyse, making it difficult to study this phenomenon by proteomics. In contrast, Evo3A exhibits a much higher filament fraction that survived prolonged ampicillin treatment (Fig. 1b). Ampicillin treatment leads to the generation of hydroxyl radicals (OH^·^) that cause DNA damage (26, 39) (Fig. 4g), which then induces the SOS response and eventually cell filamentation (14). Because SOS response is one of the processes regulated by cAMP (40, 41), and the enzyme required for the formation of cAMP is mutated in Evo3A (*cyaA* gene), it is not surprising that Evo3a has a perturbed SOS network. Besides, we have shown from our proteomics data that RecA protein, which is one of the key players that induce SOS response following DNA damage, has an increasing expression profile during the antibiotic treatment when the cells are filamenting (Fig. 3c and d).

Our data also show that filamentation is only one of the strategies for the cells to survive ampicillin treatment. After 5 h of ampicillin treatment, we also observed increased expression of proteins known to be involved in the increased tolerance of E. coli to ampicillin, such as peptidoglycan-associated lipoprotein (3), outer membrane protein TolC (42), multidrug efflux pump subunit AcrA (43), sigma factor RpoS (44), and cAMP-activated global transcriptional regulator cAMP receptor protein (CRP).

Bactericidal antibiotics, including ampicillin, have been shown to damage cells through the accumulation of reactive oxygen species (ROS), particularly hydroxyl radicals (OH^·^) (21, 34, 37). Indeed, from our proteomics data we observed the modulation of pathways related to the oxidative stress response in Evo3A when treated with ampicillin (Fig. 4c and d). We measured both the endogenous ROS generation and the mutagenesis rate in ampicillin-treated Evo3A and WT E. coli, and we observed that Evo3A has much less ROS production and a lower mutagenesis rate than the WT due to a lower OH^·^ level during ampicillin treatment (Fig. 4e to g). This suggests that Evo3A has a lower level of DNA damage than the WT. Similar observations were also reported by Molina-Quiroz et al. (13), in which a set of differentially expressed genes (compared to the WT) contributing to oxidative stress response was detected for a Δ*cyaA* mutant that exhibited an increased persister fraction.

Brynildsen and colleagues have previously constructed a metabolic model to estimate the ROS production in E. coli, starting with a previously developed metabolic reconstruction and adding 266 new ROS-producing reactions (45). Using flux balance analysis, they were able to calculate the levels of H_2_O_2_ and O_2_^−^ in response to genetic perturbations. By doing so, they predicted a set of genes that could increase ROS production when deleted (see Fig. S3 in the supplemental material). They suggested that these genes should serve as good adjuvant targets for antibiotics, as amplifying endogenous ROS production will compromise the cells’ ability to cope with the oxidative stress caused by the antibiotic, and hence enable the antibiotic to kill the cells more effectively. Interestingly, out of the genes that were predicted to increase H_2_O_2_ levels when deleted, almost all the corresponding proteins are either upregulated or newly detected in Evo3A during antibiotic treatment (Fig. S3 and Table S7), or at least not differentially expressed. The upregulation of these proteins could be understood as the mechanism of Evo3A to survive ampicillin treatment by coping with oxidative stress. The only gene in which the protein expression is downregulated is *pta*, expressing phosphate acetyltransferase. Indeed, Brynildsen et al. had experimentally checked the susceptibility of a Δ*pta* mutant *in vitro*, and it has reduced susceptibility only to fluoroquinolone, but not ampicillin (although both antibiotics generate oxidative stress) (45). The surprisingly good agreement of our data with the *in silico* prediction of anti-ROS genes further lends credence to our belief that the suppression of endogenous ROS level is a key mechanism for Evo3A filaments to survive prolonged antibiotic treatment.

10.1128/mSystems.00462-20.3 FIG S3 Protein expression of the genes in which deletions are predicted to increase H_2_O_2_ levels is either not differential or upregulated in Evo3A during ampicillin treatment. Download FIG S3, TIF file, 0.3 MB. Copyright © 2020 Sulaiman and Lam. 2020 Sulaiman and Lam This content is distributed under the terms of the Creative Commons Attribution 4.0 International license.

**FIG 7:**
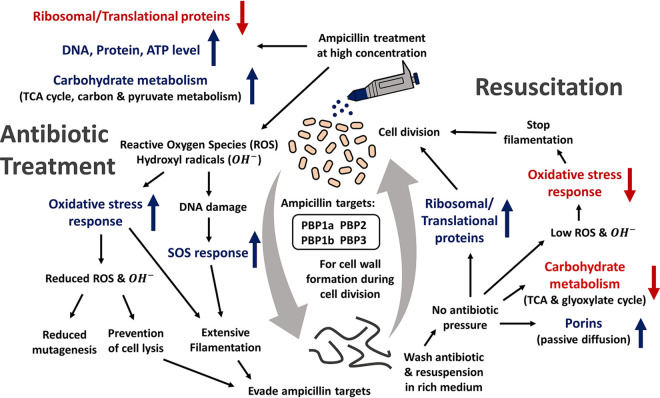
Mechanism of the filamentous persisters during antibiotic treatment and resuscitation. Upon ampicillin treatment, the cells downregulate the expression of ribosomal proteins while upregulating the expression of proteins involved in carbohydrate metabolism. The DNA, protein, and ATP contents are also increased. Ampicillin treatment led to an increased ROS and hydroxyl radical production, which is counterbalanced by an oxidative stress response in the filaments to prevent cell lysis. At the same time, the oxidative stress also causes DNA damage which then induces SOS response (marked by the increasing expression of RecA) and, in turn, filamentation. Filamentation likely serves as one of their survival strategies, as it minimizes the need for cell wall synthesis, the target of ampicillin. During resuscitation, the cells upregulate porins, allowing passive diffusion of the trace antibiotics, and also allowing ribosomal proteins to get ready for cell division. Several carbohydrate metabolism processes are downregulated, while the expression of ribosomal proteins is upregulated. The cells also have reduced oxidative stress response, thereby returning the cells to a normal state to enable cell division.

Because of the unique filamentous morphology of the persisters from Evo3A, we were able to isolate the filaments from the newly divided cells after resuspension in fresh medium (Fig. 5a and b). As shown in Fig. 5g, before cell division, the enriched filaments mainly upregulate ribosomal proteins and elongation factor Ts (EF-Ts), which implies an increase in protein synthesis. In addition, they upregulate several porins (OmpC, OmpN, and OmpF), which allows passive diffusion of small hydrophilic antibiotics, such as β-lactams, through the outer membrane (46). As opposed to those during antibiotic treatment, the enriched filaments downregulate proteins for ATP generation and carbohydrate metabolism, including proteins that play a role in the TCA cycle and glyoxylate cycle. Six out of the 15 downregulated proteins in the enriched filaments are actually those with an increasing expression profile during the antibiotic treatment, while 10 out of the 13 upregulated proteins on the enriched filaments are those with a decreasing expression profile during the antibiotic treatment. This seemingly opposite expression profile between the antibiotic treatment phase and the resuscitation phase can be explained by the simple fact that cells after resuscitation should become “normal” cells again and therefore should have a similar proteome profile as the cells before antibiotic treatment. In other words, processes that are upregulated during antibiotic treatment should be downregulated during resuspension in fresh medium, while processes that are downregulated during treatment should be upregulated during resuspension in fresh medium. This is also shown on the ATP measurements of Evo3A after 3 h of resuscitation (Fig. 5i), where we observed that the cells after 3 h of resuspension in fresh medium have similar ATP levels as the cells before ampicillin treatment. This indicated that the filamentous cells have apparently built up a reserve of ATP during the antibiotic treatment, perhaps to prepare for the subsequent resuscitation when the antibiotic stress is removed. Based on our experimental results, we proposed a summary on the mechanism employed by the filamentous persisters on Evo3A during ampicillin treatment and resuscitation (Fig. 7).

We also observed that after 3 h of resuspension in fresh medium, ElaB is one of the most upregulated proteins in the filaments but it is downregulated when the filaments divide to new progenies. We therefore hypothesize that ElaB might be an important protein that controls resuscitation and the resumption of cell division to produce new progenies. To test our hypothesis, we studied the Δ*elaB* knockout mutant and observed that knockout of *elaB* increased persistence and complementation of *elaB* reduced persistence on WT E. coli (Fig. 6a and b), in agreement with the report by Guo et al. (31). Interestingly, we also found that during resuscitation on fresh medium, there are small colony variants (SCVs) on Δ*elaB* cells (Fig. 6c and Fig. S2c), which should come from the persisters that survived ampicillin treatment. These SCVs were not observed on the WT during resuscitation, nor on untreated WT and Δ*elaB* cells, suggesting that the SCV formation is specific to Δ*elaB* cells that have been exposed to ampicillin. Unlike genetically determined stable SCVs where the small colony size is retained upon subcultivation and is a result of genetic mutations that affect thymidine or hemin biosynthesis (47–49), the SCVs observed on the Δ*elaB* mutant after ampicillin treatment are nonstable SCVs whose small size is not a consequence of a mutation (as evident by the absence of SCVs under normal growth conditions in Fig. S2c) and is not retained upon subcultivation. Also, while the small colony size of stable SCVs is attributed to the slow growth that confers tolerance to antibiotics, nonstable SCVs are a result of a late initiation of growth (50). Naturally, nonstable SCVs occur at a low frequency, and it was known that their abundance increases after exposure to specific stress conditions. As described previously, nonstable SCVs and persisters share many similar characteristics, including their low frequency within a population, their ability to revert to normal cells, and their association with chronic infections (50–54). A study on Staphylococcus aureus SCVs provided evidence that SCVs are the product of persisters and arise from dormant bacteria (50).

Overall, our results show that the absence of ElaB causes increased persistence in WT E. coli, and among the persisters are cells that fail to resume growth normally when resuspended in fresh medium. The formation of SCVs in the resuscitating Δ*elaB* mutant after ampicillin treatment is in line with our hypothesis that ElaB should be important for the transition of persisters to the new progenies during resuscitation, as the absence of *elaB* led to defects in growth resumption. However, mechanisms of how ElaB is related to increased persistence and the formation of SCVs during resuscitation are still unknown and require further study.

## MATERIALS AND METHODS

### Bacterial strains and growth conditions.

The bacterial strains used in this study are E. coli K-12 MG1655 and Evo3A, an evolved population from a recent adaptive laboratory evolution (ALE) experiment using ampicillin antibiotic (11). Briefly, to generate Evo3A, E. coli K-12 MG1655 is used as the ancestral strain for the evolution experiment. A mid-exponential-phase culture of E. coli K-12 was exposed to 100 μg/ml ampicillin antibiotic for 3 h, and the antibiotic-containing medium was removed by washing twice in LB medium. Finally, the culture was resuspended in 3 ml fresh LB and grown overnight at 37°C. The next day, 3 μl of the overnight culture was resuspended in 3 ml fresh LB and grown to mid-exponential phase. The antibiotic treatment was repeated, and after 4 cycles of antibiotic treatment and after the tolerance phenotype was observed, we collected the new population as the evolved population (Evo3A). Evo3A has 3 single point mutations (on *ybbA*, *yhgE*, and *cyaA* genes). For gene knockout and complementation experiments, the list of strains and plasmids used is in Table S1 in the supplemental material.

10.1128/mSystems.00462-20.4 TABLE S1 Strains and plasmids used for gene knockout and complementation experiments. Download Table S1, DOCX file, 0.01 MB. Copyright © 2020 Sulaiman and Lam. 2020 Sulaiman and Lam This content is distributed under the terms of the Creative Commons Attribution 4.0 International license.

10.1128/mSystems.00462-20.5 TABLE S2 Proteins on Evo3A with increasing or decreasing profile throughout 1 h, 3 h, and 5 h of antibiotic treatment. Download Table S2, DOCX file, 0.02 MB. Copyright © 2020 Sulaiman and Lam. 2020 Sulaiman and Lam This content is distributed under the terms of the Creative Commons Attribution 4.0 International license.

10.1128/mSystems.00462-20.6 TABLE S3 Differentially expressed proteins of Evo3A after 5 h of antibiotic treatment compared to Evo3A before antibiotic treatment. Download Table S3, DOCX file, 0.02 MB. Copyright © 2020 Sulaiman and Lam. 2020 Sulaiman and Lam This content is distributed under the terms of the Creative Commons Attribution 4.0 International license.

10.1128/mSystems.00462-20.7 TABLE S4 Newly detected proteins during 5 h after ampicillin treatment. Download Table S4, DOCX file, 0.02 MB. Copyright © 2020 Sulaiman and Lam. 2020 Sulaiman and Lam This content is distributed under the terms of the Creative Commons Attribution 4.0 International license.

10.1128/mSystems.00462-20.8 TABLE S5 Differentially expressed proteins of Evo3A after 1 h, 2 h, and 3 h of resuspension in fresh medium compared to those before resuspension (0 h). Download Table S5, DOCX file, 0.03 MB. Copyright © 2020 Sulaiman and Lam. 2020 Sulaiman and Lam This content is distributed under the terms of the Creative Commons Attribution 4.0 International license.

10.1128/mSystems.00462-20.9 TABLE S6 Differentially expressed proteins of enriched filaments after 3 h of resuspension in fresh medium compared to those before resuspension. Download Table S6, DOCX file, 0.02 MB. Copyright © 2020 Sulaiman and Lam. 2020 Sulaiman and Lam This content is distributed under the terms of the Creative Commons Attribution 4.0 International license.

10.1128/mSystems.00462-20.10 TABLE S7 List of genes whose deletions were predicted *in silico* to increase ROS H_2_O_2_ production levels, with the associated protein expression profile from our proteomics data. Download Table S7, DOCX file, 0.01 MB. Copyright © 2020 Sulaiman and Lam. 2020 Sulaiman and Lam This content is distributed under the terms of the Creative Commons Attribution 4.0 International license.

For our experiments, mid-exponential-phase cultures were prepared by incubating a 1:1,000-diluted overnight culture in Luria-Bertani (LB) broth at 37°C at 220 rpm until an *A*_600_ of 0.7 was reached. LB agar was used for colony counts.

### Tolerance and resistance assays.

To test the viability of Evo3A and E. coli K-12 under antibiotic treatment in exponential phase, the number of survivors after 3 h of ampicillin (125 μg/ml), ofloxacin (5 μg/ml), or kanamycin (150 μg/ml) treatment was counted by serially diluting cultures in LB broth and plating 100 μl on LB agar and spread plates. Ampicillin is a β-lactam antibiotic, and ofloxacin is a fluoroquinolone, while kanamycin is an aminoglycoside. The MICs for the population of ampicillin, ofloxacin, and kanamycin were recorded by broth macrodilution method. The MIC was determined by incubating freshly inoculated cultures in LB broth overnight with various concentration of antibiotics and observing inhibition of growth based on lack of turbidity (55). The measured MICs of ampicillin, ofloxacin, and kanamycin were 8 μg/ml, 0.125 μg/ml, and 8 μg/ml, respectively, both on WT and on Evo3A.

### DNA, protein, and ATP measurement.

Mid-exponential-phase Evo3A was treated with ampicillin (125 μg/ml) at *t* = 0 h, 1 h, 3 h, and 5 h after ampicillin treatment. Then, 500 μl of cells was taken for DNA and protein concentration measurement, and 100 μl of cells was taken for ATP measurement at each time point. Total DNA from Evo3A before and after 1, 3, and 5 h of ampicillin treatment was extracted using Qiagen’s DNeasy blood and tissue kit according to the manufacturer’s protocol. When extracting the DNA, 4 μl of RNase A (100 mg/ml) was added. The concentration of DNA was measured using a NanoVue Plus spectrophotometer (GE Healthcare). For protein concentration determination, cells were suspended in 250 μl of lysis buffer (0.5% SDS, 50 mM Tris-HCl, pH 8.0), incubated at 95°C for 5 min, and sonicated for 3 min. The protein concentration was determined using the bicinchoninic acid (BCA) protein assay (Pierce BCA Protein assay kit), and bovine serum albumin (BSA) was used to generate the standard curve. For ATP measurements, BacTiter-Glo assay (Promega) was used according to the manufacturer’s protocol. Mid-exponential Evo3A cells were treated with ampicillin (125 μg/ml), and at *t* = 0 h, 1 h, 3 h, and 5 h after ampicillin treatment, 100 μl of aliquots was taken from the culture and mixed with 100 μl of BacTiter-Glo reagents. After incubation for 5 min, the luminescence, which is proportional to the amount of ATP present in the sample, was recorded. A standard curve with known ATP concentrations (0, 0.125, 0.25, 0.5, and 1 μM) was prepared.

### Epifluorescence microscopy.

Epifluorescence microscopy was used to observe the population-wise behavior of Evo3A and E. coli K-12 upon antibiotic treatment and resuscitation. Five hundred microliters of the cells being treated with antibiotic were taken, centrifuged, and resuspended in 0.85% NaCl. Cultures were stained with the LIVE/DEAD *Bac*Light bacterial viability kit (Molecular Probes) according to the manufacturer’s standard protocol. A 1.5-μl amount of dye mixture containing SYTO 9 (1.67 mM) and propidium iodide (10 mM) was added to the 500-μl culture and incubated in the dark for 10 min. Stained cells were viewed with a fluorescence microscope (Eclipse Ni-U upright microscope) with appropriate filter sets. Images were captured with Nikon DS-Fi3 and associated software (NIS-Elements Ver. 5.00).

### Microscopic observation of recovering filaments on agarose gel pads.

Time-lapse microscopy was used to observe the filaments on Evo3A upon resuscitation after antibiotic treatment. For time-lapse microscopy, LB pads with 1% agarose were made by dissolving 1 g of ultrapure agarose in 100 ml of fresh LB medium using a microwave. Our preparation method was adopted from the one described by Young et al. with a slight modification (56). LB-agarose was microwaved for ∼1 min, until the liquid was boiling. Three glass slides were arranged side by side with their edges touching, and two more glass slides were placed on top of the first three in a staggered configuration. The space between the two glass slides sitting on the top was for the LB pads. Approximately 1 ml of LB-agarose was put into the center well between the two upper glass slides, and another glass slide was placed on top of the molten LB-agarose to control the thickness of the pads. The LB-agarose was allowed to harden for ∼45 min at room temperature. Then, the glass slide on the top was removed and the LB-agarose was cut into smaller-size squares (1.5 cm by 1.5 cm) using a sterile spatula.

After treating Evo3A with a lethal dose of ampicillin (125 μg/ml) for 5 h, the surviving cells were harvested at 7,000 × *g* for 4 min, washed with fresh LB medium twice to remove the antibiotic, and then resuspended with 3 ml of LB medium. The culture was further diluted 1:5 in fresh LB to reduce the cell density. Fifteen microliters of this diluted sample was spotted onto a glass slide, and the LB pad was placed on top of the bacteria. Then, a small coverslip (22 mm by 22mm) was placed over the LB pad. The microscope used was a fully motorized Nikon Ti-E with Perfect Focus System. The objective used was a Plan Fluor phase contrast 40× dry objective with 0.6 numerical aperture (NA). An environmental control chamber mounted in a fully motorized XY stage was used to keep the samples at 37°C during the course of the experiment (Chamlide TC temperature, humidity, and CO_2_ chamber). Images were taken every 10 min for 3 h with an Andor Zyla ultra-low noise scalable complementary metal oxide semiconductor (sCMOS) camera (2,408 by 2,048 pixels) and associated software (MetaMorph Premier 7.8.6; Molecular Devices). The resulting images were processed with ImageJ (57) to remove background noise.

### Determination of changes in the frequency of mutagenesis.

To determine the changes in the frequency of mutagenesis, we calculated the change in the generation of rifampin-resistant mutants. Mid-exponential-phase cultures of Evo3A and WT were challenged with ampicillin (125 μg/ml) for 1 h, washed three times with fresh LB, and grown overnight to enrich for rifampin-resistant mutants. After 24 h of growth, each culture was plated on plates of LB agar and LB agar supplemented with rifampin at 100 μg/ml as previously reported (58). The frequency of mutagenesis was calculated by dividing the total number of rifampin-resistant cells by the total number of cells.

### Fluorometric detection of intracellular ROS.

E. coli K-12 (WT) and Evo3A were grown to mid-exponential phase and exposed to ampicillin (125 μg/ml) for 1 h. As a positive control, the cells were treated with 50 μM FeSO_4_ and 10 mM H_2_O_2_ for 1 h to generate hydroxyl radicals by the Fenton reaction as previously described (26). Fluorescent probe H_2_DCFDA (dihydrodichlorofluorescein diacetate) was used to assess the total ROS level in the cells (59). Cultures were washed twice with phosphate-buffered saline (PBS, 1×, pH 7.4) and incubated for 30 min in the presence of 50 μM H_2_DCFDA (prepared in dimethyl sulfoxide). After washing twice with 1× PBS, cells were disrupted by sonication for 4 min and then centrifuged for 7 min at 13,000 × *g* to remove cell debris. Fluorescence intensity was determined in a Varioskan LUX multimode microplate reader (Thermo Scientific) (excitation, 410 nm; emission, 519 nm).

### Protein localization and complementation.

Localization of ElaB using GFP was conducted following the protocol from a previous study (31). Three microliters of overnight cultures harboring pCA24N-*elaB-gfp* was inoculated into 3 ml fresh LB broth containing chloramphenicol (30 μg/ml) until an *A*_600_ of 1 was reached. Isopropyl-β-d-thiogalactopyranoside (IPTG; 0.5 mM) was added to induce ElaB-GFP expression for 2 h before imaging. Here, we captured an image of filaments, which were rare in this population, to show the localization of ElaB on filamentous cells.

For complementation of *elaB*, 3 μl of overnight Δ*elaB* cultures harboring pCA24N-*elaB* was inoculated into 3 ml fresh LB broth until the turbidity at 600 nm reached ∼0.3. WT and Δ*elaB* strains harboring empty pCA24N plasmid were used as controls. Then, 1 mM IPTG was added to induce ElaB expression for 2 h. For survival measurement, ampicillin (125 μg/ml) was added to both the Δ*elaB* strain harboring pCA24N-*elaB* and pCA24N only, and also WT harboring pCA24N only, for 3 h. The survivors were counted by serially diluting cultures in LB broth and plating 100 μl on LB agar and spread plates.

### Enrichment of filaments for proteomics analysis.

A mid-exponential-phase culture of Evo3A was exposed to 125 μg/ml ampicillin for 5 h. After 5 h, the antibiotic-containing medium was removed by washing twice in LB medium, and the cell pellet was resuspended in 3 ml of LB medium and grown at 37°C, 220 rpm, for 3 h. After 3 h of resuspension in fresh medium, there will be a mixture of filaments (measured to be >5 μm) and normal-sized bacteria (new progeny after cell division, measured to be <5 μm). The cell culture was passed through a polyvinylidene fluoride membrane filter (Merck Millipore) with a pore size of 5 μm. After filtration, the filaments were retained on the membrane, and the new progeny passed through the filter as the filtrate. The filtrate was collected in a microcentrifuge tube and centrifuged at 7,000 × *g* for 4 min, while the retentate was washed from the filter using an 0.85% NaCl solution, collected in a microcentrifuge tube, and centrifuged at 7,000 × *g* for 4 min.

### Sample preparation for proteomics analysis.

For time course proteomics analysis during antibiotic treatment, Evo3A was treated with ampicillin (125 μg/ml), and then aliquots were taken after 1, 3, and 5 h of treatment from the same replicate. The control sample was untreated Evo3A. To remove the intact dead cells after antibiotic treatment and enrich for the persisters for proteomics analysis, we employed our previously developed magnetic beads-based separation method (3). For time course proteomics analysis during resuscitation, Evo3A after 5 h of ampicillin treatment was resuspended in fresh LB medium, and then aliquots were taken after 1, 2. and 3 h of growth from the same replicate. The control sample was Evo3A after 5 h of ampicillin treatment, but not yet resuspended into the fresh LB medium. For proteomics analysis of the enriched filaments and the new progeny after resuspension in fresh medium, Evo3A after 5 h of ampicillin treatment was resuspended in fresh LB medium and taken after 3 h of growth. The filaments were separated from the new progeny by using a PVDF membrane as previously described. For all proteomics experiments, three biological replicates were performed for each sample including the control sample, and two technical replicates were performed for each biological replicate.

The cell pellet was suspended in 300 μl of lysis buffer (0.5% SDS, 50 mM Tris-HCl, pH 8.0, and 25 mM dithiothreitol), incubated at 95°C for 5 min, and sonicated for 3 min. The sample was centrifuged (16,000 × *g* for 5 min) to remove cell debris and insoluble materials. An aliquot of the sample was taken for BCA protein assay (Pierce BCA protein assay kit). For shotgun proteomics, 200 μg of proteins was mixed with 250 μl of the exchange buffer (6 M urea, 50 mM Tris-HCl, pH 8.0, 600 mM guanidine HCl), transferred to an Amicon filter device (Millipore, Darmstadt, Germany), and centrifuged (14,000 × *g* for 20 min). Then, 250 μl of the exchange buffer was added once again to the filter device and centrifuged, and the filtrate in the collection tube was discarded. The proteins in the filter device were alkylated with iodoacetamide (IAA; 50 mM in exchange buffer) in the dark for 20 min and then centrifuged (14,000 × *g* for 20 min). Two hundred fifty microliters of the exchange buffer was added to the filter device and centrifuged, and the filtrate in the collection tube was discarded. To dilute the urea concentration, 250 μl of 50 mM ammonium bicarbonate was added to the filter device and centrifuged (14,000 × *g* for 20 min). This step was repeated once. Proteins were digested by sequencing-grade modified trypsin (1:100 [wt/wt]; Promega, Madison, WI) for 10 h at 37°C. Then, the sample was acidified with 10% formic acid to a final concentration of 0.1% (vol/vol) and centrifuged for 16,000 × *g* for 5 min. Finally, the samples were desalted with a C_18_ reverse-phase ZipTip (Millipore, Darmstadt, Germany), dried with a SpeedVac (Eppendorf, Hamburg, Germany) for 15 min, and stored at −20°C before use.

### Liquid chromatography-tandem mass spectrometry (LC-MS/MS).

We adopted standard shotgun proteomics techniques (60) to process the sample through the Thermo Scientific Accela high-performance liquid chromatography (HPLC) system coupled to a dual cell linear ion trap MS, LTQ Velos (Thermo Fisher Scientific, Bremen, Germany), which is interfaced to a nano-electrospray ion source. Approximately 1 μg of the protein digest was injected into the HPLC system and separated on a C_18_ column (Thermo Bio-Basic-18; 150 by 0.1 mm, 300-Å pore size, 5-μm particle size) at a flow rate of 180 μl/min with a split flow before going to the valve to prevent back pressure. The mobile phase composition is 0.1% formic acid in water for solvent A and 0.1% formic acid in acetonitrile for solvent B. The gradient was applied from 2% to 10% of solvent B for 4 min, from 10% to 32% of solvent B for 60 min, from 32% to 60% of solvent B for 6 min, and then from 60% to 80% of solvent B for 2 min. At the end, the mobile phase was kept at 80% of solvent B for 2 min and then decreased to 2% of solvent B for 2 min. Fourteen minutes of equilibration with 2% of solvent B was applied before the next injection. The *m/z* range acquired in the MS full scan was 400 to 2,000 Da. The top 10 most intense precursor ions were selected to the MS^2^ by using collision-induced-dissociation (CID) with dynamic exclusion of 60 s, and the singly charged precursor ions were excluded from the MS^2^. The MS^2^ isolation width was 2.0 *m/z*, and the normalized collision energy was set at 30%.

### Sequence database searching.

The raw data acquired by the Thermo Scientific LTQ Velos were converted to mzML files by msconvert of the ProteoWizard (version 3.0.11676 64-bit) (61). The mzML files were searched using Comet (version 2016.01 rev.2) against the E. coli K-12 protein sequence database obtained from UniProt (downloaded January 2018). The sequences of common contaminants, such as trypsin and human keratins, and decoy sequences generated by shuffling amino acid sequences between tryptic cleavage sites were added to the database. The decoy sequence in the database was used for the false-discovery rate (FDR) estimation of the identified peptides. The search parameter criteria were set as follows: 3-atomic-mass-unit (amu) peptide mass tolerance, monoisotopic mass type, fully digested enzyme termini, 1.005-amu fragment bin tolerance, 0.4-amu fragment bin offset, and carbamidomethylated cysteine and oxidated methionine as the fixed and variable modifications, respectively. The search results from Comet were processed by PeptideProphet (62) and iProphet and ProteinProphet of the Trans-Proteomics Pipeline (TPP) (63) in the decoy-assisted nonparametric mode. Every mzML run was analyzed independently. Protein identifications were filtered at a false-discovery rate of 0.01 as predicted by ProteinProphet.

### Label-free quantification by spectral counting.

The quantification of proteins was given by the normalized spectral abundance factor (NSAF) (64), where the number of peptide-spectrum matches (PSMs) for each protein divided by the length of the corresponding protein is normalized to the total number of PSMs divided by the lengths of all identified proteins. Only proteins with average spectral counts (across all runs) of at least 3 were considered for quantification. Two different approaches were employed to screen for potentially important proteins: (i) a before-and-after time point comparison and (ii) a time-series analysis during antibiotic treatment to look for trends.

For the first approach, the proteome of Evo3A after 5 h of antibiotic treatment was compared to the one before antibiotic treatment. During the resuscitation phase, a similar before-and-after comparison was made to compare the proteome of the filaments after 3 h of resuspension to the that of the filaments before resuspension, and also to compare the proteome of the new progeny (after division) to the filaments (before division). The search results from the two technical replicates of each biological replicate were combined, and the proteins identified in at least two out of three biological replicates were used for label-free quantification by spectral counting. Student’s *t* test was employed on the NSAF values to detect differential expression between the two time points. Benjamini-Hochberg (BH) multiple testing correction was applied to the *t* test *P* values to control the false-discovery rate (FDR) at 10% (65). To further reduce false discoveries and limit our attention to the more highly regulated proteins, only proteins with fold changes higher or lower than ±1.5-fold were considered differentially expressed in our subsequent analysis. Moreover, the newly detected proteins on Evo3A after antibiotic treatment (not identified before antibiotic treatment), and also the newly detected proteins after resuscitation (not identified before resuspension in fresh medium), are also retained for analysis, as they are also likely to have increased expression during the antibiotic treatment or the resuscitation process. To minimize false positives, we further limit our attention to only newly detected proteins with spectral counts greater than 5. Here, we assume that these newly detected proteins with sufficiently high spectral counts are also upregulated during the antibiotic treatment and/or resuscitation. The differentially expressed proteins and newly detected proteins are subjected to pathway-enrichment study (KEGG database) and functional classification (GO analysis classified by biological process) by the DAVID (Database for Annotation, Visualization and Integrated Discovery) algorithm version 6.8 (19). The *P* value threshold and protein count threshold for both pathway enrichment and functional classification were set to 0.1 and 2, respectively.

For the second approach, a time series analysis was used to spot any increasing or decreasing trend of the protein expression profiles in Evo3A during the antibiotic treatment. For each of the biological replicates, the NSAF values for every protein after 1 h, 3 h, and 5 h of antibiotic treatment were divided by the NSAF value before antibiotic treatment (0 h) to obtain the log_2_(fold change) of the protein. Because there are three time points and three biological replicates, nine data points are generated for each protein which are then fitted to a linear regression model on Matlab (R2014b). Proteins that have significant slopes (different from zero) with a *P* value below 0.05 or an absolute log_2_(*P* value) above 4.32 are retained; these proteins should have an increasing or decreasing profile from 1 h to 5 h of antibiotic treatment. To highlight potentially important proteins among the proteins with increasing or decreasing expression profiles during antibiotic treatment, STRING v10.5 (66) was used to predict the protein-protein interactions and to visualize the interaction network.

### Data availability.

The mass spectrometry proteomics data have been deposited to ProteomeXchange (67) via the PRIDE (68) repository with the data set identifier PXD016271. Whole-genome sequence data of Evo3A have been deposited in the BioSample database under the accession number SAMN11269515.
