# Supplementary figures and images for: Proteomic Study of the Survival and Resuscitation Mechanisms of Filamentous Persisters in an Evolved Escherichia coli Population from Cyclic Ampicillin Treatment
Source: mSystems. 2020 Jul 28;5(4):e00462-20. doi: 10.1128/mSystems.00462-20 (PMC7394356; doi:10.1128/mSystems.00462-20)

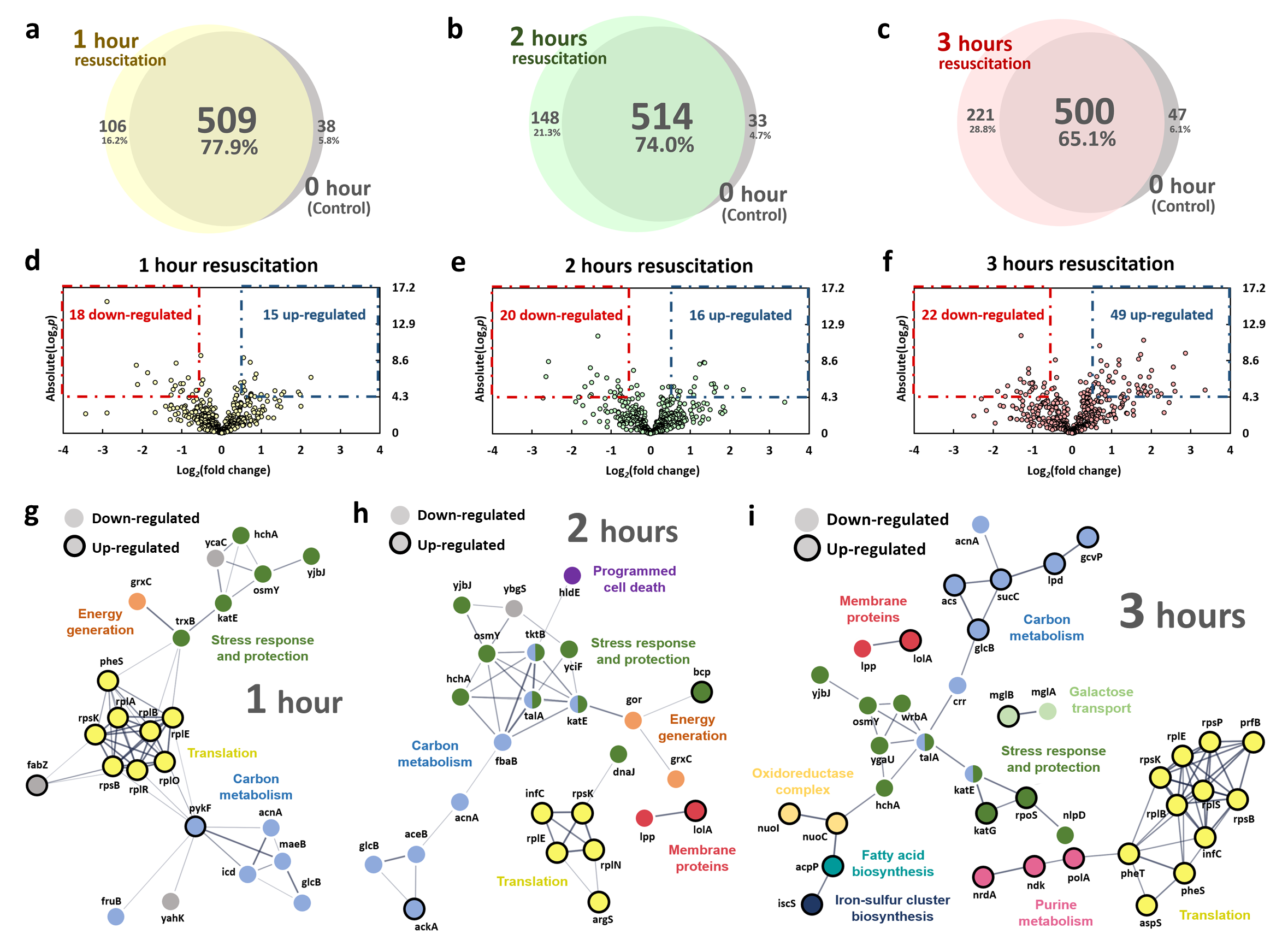

Supplement: FIG S1 [file mSystems.00462-20-sf001.tif]

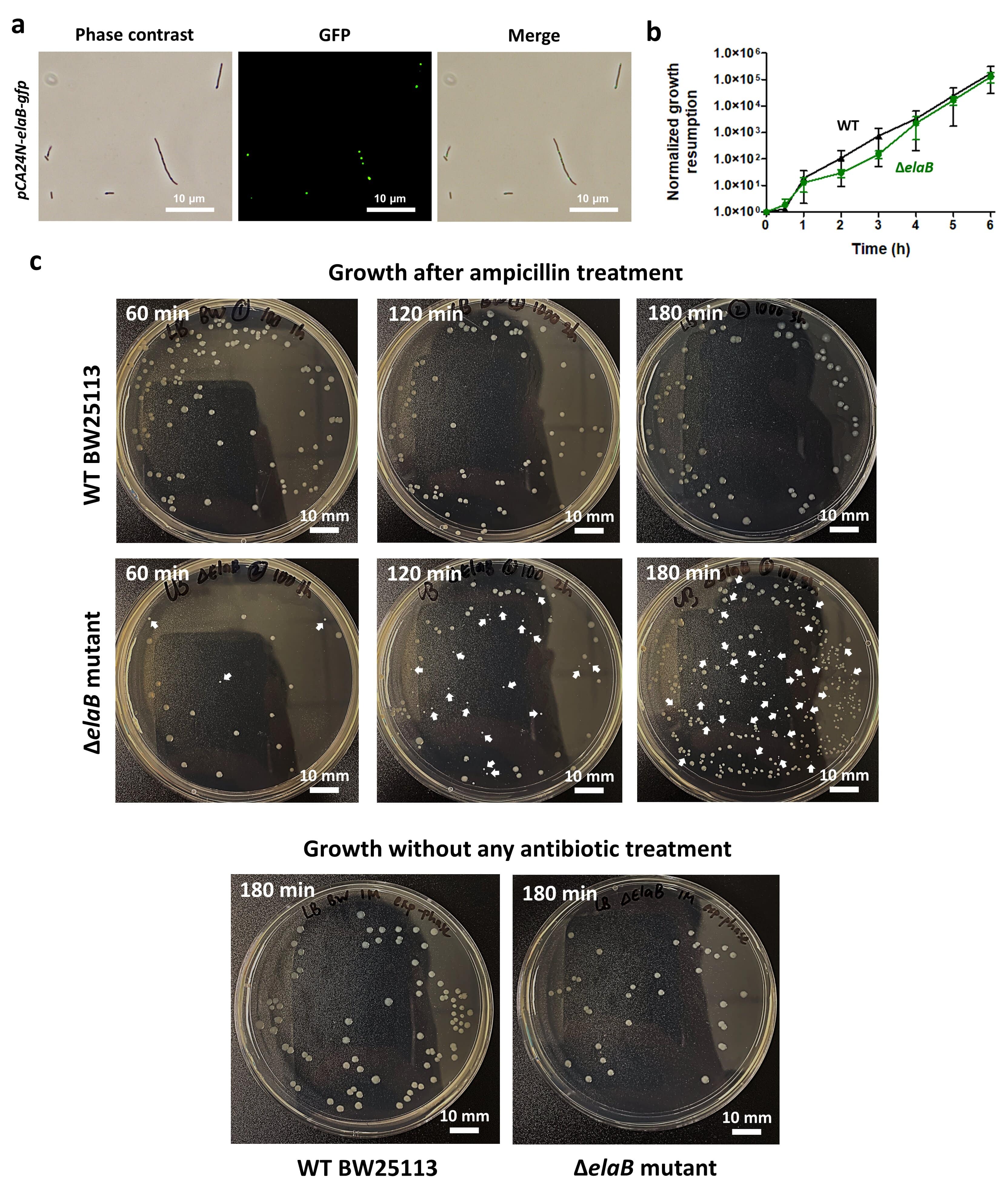

Supplement: FIG S2 [file mSystems.00462-20-sf002.jpg]

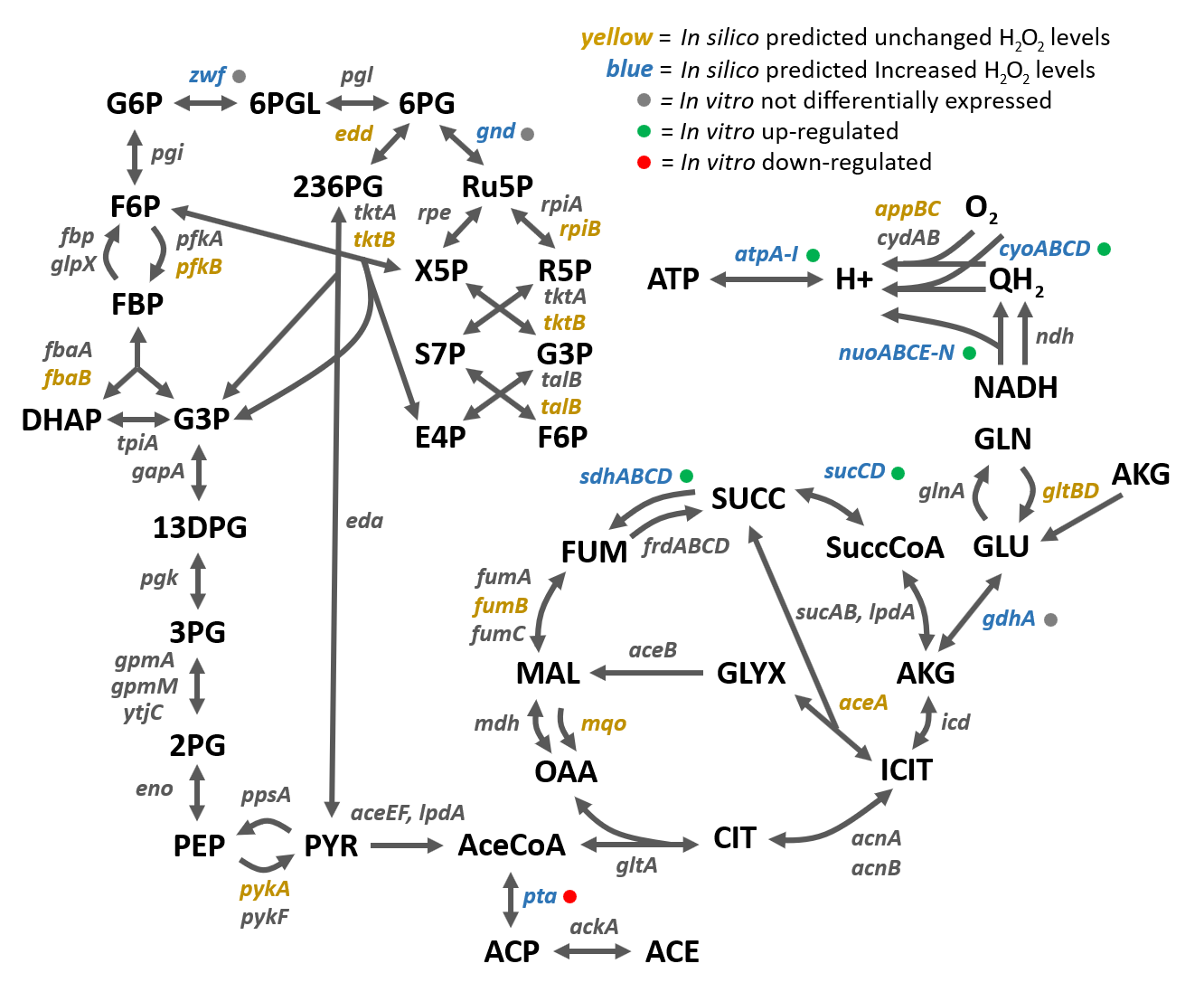

Supplement: FIG S3 [file mSystems.00462-20-sf003.tif]
