# Supplementary material for: Proteomic Study of the Survival and Resuscitation Mechanisms of Filamentous Persisters in an Evolved Escherichia coli Population from Cyclic Ampicillin Treatment
Source: mSystems. 2020 Jul 28;5(4):e00462-20. doi: 10.1128/mSystems.00462-20 (PMC7394356; doi:10.1128/mSystems.00462-20)
Supplement: TABLE S1 [file mSystems.00462-20-st001.docx]

| **Strain or plasmid** | **Description** | **ref.** |
| --- | --- | --- |
| *E. coli* K-12 |  |  |
| BW25113 (WT) | K-12 derivative: ΔaraBAD, ΔrhaBAD | (1) |
| Δ*elaB* mutant | BW25113 derivative: Δ*elaB748*::*kan* | (1) |
|  |  |  |
| Plasmids |  |  |
| pCA24N | Cm^r^ *lacl*^q^ | (2) |
| pCA24N-*elaB* | Cm^r^ *lacl*^q^ P*_T5-lac_*::*elaB* | (2) |
| pCA24N-*elaB-gfp* | Cm^r^ *lacl*^q^ P*_T5-lac_*::*elaB*, with *gfp* gene | (2) |

*****Cm^r^ indicate chloramphenicol resistance. P indicate promoter.

**References**

1. Baba T, Ara T, Hasegawa M, Takai Y, Okumura Y, Baba M, Datsenko KA, Tomita M, Wanner BL, Mori H. 2006. Construction of *Escherichia coli* K‐12 in‐frame, single‐gene knockout mutants: the Keio collection. Mol Syst Biol 2:2006.0008.

2. Kitagawa M, Ara T, Arifuzzaman M, Ioka-Nakamichi T, Inamoto E, Toyonaga H, Mori H. 2005. Complete set of ORF clones of *Escherichia coli* ASKA library (A Complete S et of *E. coli* K-12 ORF A rchive): Unique Resources for Biological Research. DNA Res 12:291-299.
