# Supplementary material for: Proteomic Study of the Survival and Resuscitation Mechanisms of Filamentous Persisters in an Evolved Escherichia coli Population from Cyclic Ampicillin Treatment
Source: mSystems. 2020 Jul 28;5(4):e00462-20. doi: 10.1128/mSystems.00462-20 (PMC7394356; doi:10.1128/mSystems.00462-20)
Supplement: TABLE S2 [file mSystems.00462-20-st002.docx]

| **UniProt ID** | **p-value** | **Slope** | **Gene** | **Protein name** |
| --- | --- | --- | --- | --- |
| P0ADG4 | 0.0003 | -0.80 | *suhB* | Inositol-1-monophosphatase |
| P68066 | 0.0014 | -0.65 | *grcA* | Autonomous glycyl radical cofactor |
| P36672 | 0.0237 | -0.64 | *treB* | PTS system trehalose-specific EIIBC component |
| P0ADG7 | 0.0033 | -0.58 | *guaB* | Inosine-5'-monophosphate dehydrogenase |
| P0ADZ0 | 0.0347 | -0.55 | *rplW* | 50S ribosomal protein L23 |
| P28904 | 0.0118 | -0.55 | *treC* | Trehalose-6-phosphate hydrolase |
| P0A7E5 | 0.0133 | -0.46 | *pyrG* | CTP synthase |
| P0C018 | 0.0035 | -0.41 | *rplR* | 50S ribosomal protein L18 |
| P0A7W7 | 0.0036 | -0.41 | *rpsH* | 30S ribosomal protein S8 |
| P60624 | 0.0023 | -0.41 | *rplX* | 50S ribosomal protein L24 |
| P0AG48 | 0.0327 | -0.39 | *rplU* | 50S ribosomal protein L21 |
| P38489 | 0.0499 | -0.37 | *nfsB* | Oxygen-insensitive NAD(P)H nitroreductase |
| P02931 | 0.0194 | -0.37 | *ompF* | Outer membrane porin F |
| P06959 | 0.0060 | -0.36 | *aceF* | Dihydrolipoyllysine-residue acetyltransferase component of pyruvate dehydrogenase complex |
| P0A7K6 | 0.0377 | -0.34 | *rplS* | 50S ribosomal protein L19 |
| P33602 | 0.0164 | -0.33 | *nuoG* | NADH-quinone oxidoreductase subunit G |
| P0AFG8 | 0.0026 | -0.32 | *aceE* | Pyruvate dehydrogenase E1 component |
| P21888 | 0.0093 | -0.32 | *cysS* | Cysteine--tRNA ligase |
| P0A9M8 | 0.0241 | -0.32 | *pta* | Phosphate acetyltransferase |
| P02358 | 0.0112 | -0.31 | *rpsF* | 30S ribosomal protein S6 |
| P0A7V0 | 0.0012 | -0.30 | *rpsB* | 30S ribosomal protein S2 |
| P0A7L0 | 0.0199 | -0.29 | *rplA* | 50S ribosomal protein L1 |
| P0A7T7 | 0.0015 | -0.29 | *rpsR* | 30S ribosomal protein S18 |
| P02413 | 0.0251 | -0.28 | *rplO* | 50S ribosomal protein L15 |
| P0AG55 | 0.0016 | -0.27 | *rplF* | 50S ribosomal protein L6 |
| P04949 | 0.0226 | -0.26 | *fliC* | Flagellin |
| P68679 | 0.0044 | -0.25 | *rpsU* | 30S ribosomal protein S21 |
| P76177 | 0.0478 | -0.25 | *ydgH* | Protein YdgH |
| P61175 | 0.0191 | -0.25 | *rplV* | 50S ribosomal protein L22 |
| P06996 | 0.0311 | -0.24 | *ompC* | Outer membrane porin C |
| P64604 | 0.0446 | -0.23 | *mlaD* | Intermembrane phospholipid transport system binding protein MlaD |
| P00959 | 0.0171 | -0.23 | *metG* | Methionine--tRNA ligase |
| P0A8A0 | 0.0495 | -0.23 | *yebC* | Probable transcriptional regulatory protein YebC |
| P0A7Z4 | 0.0028 | -0.23 | *rpoA* | DNA-directed RNA polymerase subunit alpha |
| P0A7J7 | 0.0061 | -0.23 | *rplK* | 50S ribosomal protein L11 |
| P0A7V8 | 0.0114 | -0.18 | *rpsD* | 30S ribosomal protein S4 |
| P02359 | 0.0052 | -0.18 | *rpsG* | 30S ribosomal protein S7 |
| P0A7K2 | 0.0158 | -0.15 | *rplL* | 50S ribosomal protein L7/L12 |
| P0A6P1 | 0.0259 | -0.13 | *tsf* | Elongation factor Ts (EF-Ts) |
| P0A8T7 | 0.0300 | -0.13 | *rpoC* | DNA-directed RNA polymerase subunit beta' |
| P0A7R5 | 0.0005 | -0.13 | *rpsJ* | 30S ribosomal protein S10 |
| P0AG67 | 0.0229 | -0.10 | *rpsA* | 30S ribosomal protein S1 |
| P0ABB0 | 0.0252 | 0.12 | *atpA* | ATP synthase subunit alpha |
| P0AFG6 | 0.0097 | 0.13 | *sucB* | Dihydrolipoyllysine-residue succinyltransferase component of 2-oxoglutarate dehydrogenase complex |
| P0A8L1 | 0.0421 | 0.17 | *serS* | Serine--tRNA ligase |
| P0A7G6 | 0.0164 | 0.18 | *recA* | Protein RecA |
| P0A8M3 | 0.0394 | 0.22 | *thrS* | Threonine--tRNA ligase |
| P61889 | 0.0025 | 0.23 | *mdh* | Malate dehydrogenase |
| P0AFH8 | 0.0033 | 0.26 | *osmY* | Osmotically-inducible protein Y |
| P0ABH7 | 0.0031 | 0.27 | *gltA* | Citrate synthase |
| P00509 | 0.0314 | 0.30 | *aspC* | Aspartate aminotransferase |
| P17169 | 0.0416 | 0.33 | *glmS* | Glutamine--fructose-6-phosphate aminotransferase |
| P77804 | 0.0389 | 0.35 | *ydgA* | Protein YdgA |
| P0ADZ7 | 0.0046 | 0.37 | *yajC* | Sec translocon accessory complex subunit YajC |
| P0AFF6 | 0.0103 | 0.40 | *nusA* | Transcription termination/antitermination protein NusA |
| P0ABT2 | 0.0062 | 0.43 | *dps* | DNA protection during starvation protein |
| P0AE08 | 0.0047 | 0.48 | *ahpC* | Alkyl hydroperoxide reductase C |
| P0A6F3 | 0.0067 | 0.49 | *glpK* | Glycerol kinase |
| P10384 | 0.0020 | 0.56 | *fadL* | Long-chain fatty acid transport protein |
| P02925 | 0.0072 | 0.70 | *rbsB* | Ribose import binding protein RbsB |
