# Supplementary material for: Proteomic Study of the Survival and Resuscitation Mechanisms of Filamentous Persisters in an Evolved Escherichia coli Population from Cyclic Ampicillin Treatment
Source: mSystems. 2020 Jul 28;5(4):e00462-20. doi: 10.1128/mSystems.00462-20 (PMC7394356; doi:10.1128/mSystems.00462-20)
Supplement: TABLE S3 [file mSystems.00462-20-st003.docx]

| **UniProt ID** | **p-value** | **Folds** | **Gene** | **Protein name** |
| --- | --- | --- | --- | --- |
| P0ADG7 | 0.01104 | 0.15 | *guaB* | Inosine-5'-monophosphate dehydrogenase |
| P0A7W7 | 0.00108 | 0.22 | *rpsH* | 30S ribosomal protein S8 |
| P0A7E5 | 0.00214 | 0.25 | *pyrG* | CTP synthase |
| P0AD61 | 0.01290 | 0.27 | *pykF* | Pyruvate kinase I |
| P60624 | 0.00307 | 0.27 | *rplX* | 50S ribosomal protein L24 |
| P0C018 | 0.00974 | 0.28 | *rplR* | 50S ribosomal protein L18 |
| P32132 | 0.01611 | 0.30 | *typA* | GTP-binding protein TypA/BipA |
| P0A9M8 | 0.00052 | 0.33 | *pta* | Phosphate acetyltransferase |
| P02413 | 0.02245 | 0.33 | *rplO* | 50S ribosomal protein L15 |
| P06959 | 0.03194 | 0.33 | *aceF* | Dihydrolipoyllysine-residue acetyltransferase component of pyruvate dehydrogenase complex |
| P60438 | 0.01814 | 0.35 | *rplC* | 50S ribosomal protein L3 |
| P0A8A0 | 0.01042 | 0.36 | *yebC* | Probable transcriptional regulatory protein YebC |
| P0A7T7 | 0.00442 | 0.38 | *rpsR* | 30S ribosomal protein S18 |
| P0AG55 | 0.00028 | 0.39 | *rplF* | 50S ribosomal protein L6 |
| P00961 | 0.00770 | 0.40 | *glyS* | Glycine--tRNA ligase beta subunit |
| P68679 | 0.01667 | 0.40 | *rpsU* | 30S ribosomal protein S21 |
| P0A7L0 | 0.01689 | 0.44 | *rplA* | 50S ribosomal protein L1 |
| P02358 | 0.02756 | 0.45 | *rpsF* | 30S ribosomal protein S6 |
| P0A6P1 | 0.00057 | 0.45 | *tsf* | Elongation factor Ts |
| P0A7K2 | 0.00928 | 0.46 | *rplL* | 50S ribosomal protein L7/L12 |
| P0A7A9 | 0.02229 | 0.46 | *ppa* | Inorganic pyrophosphatase |
| P0A7V0 | 0.00469 | 0.48 | *rpsB* | 30S ribosomal protein S2 |
| P00959 | 0.03399 | 0.48 | *metG* | Methionine--tRNA ligase |
| P0A817 | 0.01413 | 0.49 | *metK* | S-adenosylmethionine synthase |
| P0A7J7 | 0.00447 | 0.50 | *rplK* | 50S ribosomal protein L11 |
| P0A7V8 | 0.00593 | 0.52 | *rpsD* | 30S ribosomal protein S4 |
| P06996 | 0.02645 | 0.53 | *ompC* | Outer membrane porin C |
| P0A7Z4 | 0.00279 | 0.53 | *rpoA* | DNA-directed RNA polymerase subunit alpha |
| P07813 | 0.03480 | 0.53 | *leuS* | Leucine--tRNA ligase |
| P0A8T7 | 0.00595 | 0.58 | *rpoC* | DNA-directed RNA polymerase subunit beta |
| P02359 | 0.01551 | 0.58 | *rpsG* | 30S ribosomal protein S7 |
| P0A7S9 | 0.02011 | 0.59 | *rpsM* | 30S ribosomal protein S13 |
| P0CE47 | 0.01018 | 0.59 | *tufA* | Elongation factor Tu 1 |
| P60723 | 0.00329 | 0.60 | *rplD* | 50S ribosomal protein L4 |
| P0A8V2 | 0.01671 | 0.65 | *rpoB* | DNA-directed RNA polymerase subunit beta |
| P0A799 | 0.01111 | 1.75 | *pgk* | Phosphoglycerate kinase |
| P0ADY3 | 0.03011 | 1.96 | *rplN* | 50S ribosomal protein L14 |
| P0ABA6 | 0.02476 | 2.05 | *atpG* | ATP synthase gamma chain |
| P09394 | 0.00756 | 2.36 | *glpQ* | Glycerophosphodiester phosphodiesterase |
| P22259 | 0.01126 | 2.47 | *pckA* | Phosphoenolpyruvate carboxykinase |
| P00956 | 0.00708 | 2.47 | *ileS* | Isoleucine--tRNA ligase |
| P0A6F3 | 0.00529 | 2.53 | *glpK* | Glycerol kinase |
| P61889 | 0.00199 | 2.66 | *mdh* | Malate dehydrogenase |
| P36683 | 0.00003 | 2.78 | *acnB* | Aconitate hydratase B |
| Q1PI59 | 0.03492 | 2.86 | *mdh* | Malate dehydrogenase |
| P0A8N3 | 0.01138 | 3.00 | *lysS* | Lysine--tRNA ligase |
| P0A8M3 | 0.01130 | 3.06 | *thrS* | Threonine--tRNA ligase |
| P0AC41 | 0.00650 | 3.07 | *sdhA* | Succinate dehydrogenase flavoprotein subunit |
| P0ABB4 | 0.00562 | 3.47 | *atpD* | ATP synthase subunit beta |
| P77774 | 0.00354 | 3.49 | *bamB* | Outer membrane protein assembly factor BamB |
| P0A6K6 | 0.01414 | 3.94 | *deoB* | Phosphopentomutase |
| P0AFG3 | 0.00361 | 4.28 | *sucA* | 2-oxoglutarate dehydrogenase E1 component |
| P0ADE6 | 0.01856 | 4.39 | *kbp* | Potassium binding protein Kbp |
| P0AFG6 | 0.01063 | 4.77 | *sucB* | Dihydrolipoyllysine-residue succinyltransferase component of 2-oxoglutarate dehydrogenase complex |
| P0ADZ7 | 0.01157 | 4.78 | *yajC* | Sec translocon accessory complex subunit YajC |
| P77804 | 0.00198 | 5.50 | *ydgA* | Protein YdgA |
| P13035 | 0.00927 | 5.69 | *glpD* | Aerobic glycerol-3-phosphate dehydrogenase |
| P10384 | 0.01003 | 5.86 | *fadL* | Long-chain fatty acid transport protein |
| P00509 | 0.00284 | 5.93 | *aspC* | Aspartate aminotransferase |
| P09127 | 0.02926 | 6.23 | *hemX* | Protein HemX |
| P08200 | 0.01129 | 6.82 | *icd* | Isocitrate dehydrogenase |
| P0ABH7 | 0.00071 | 7.27 | *gltA* | Citrate synthase |
| P25553 | 0.00159 | 8.21 | *aldA* | Lactaldehyde dehydrogenase |
| P0C0V0 | 0.00125 | 9.57 | *degP* | Periplasmic serine endoprotease DegP |
| P0AFH8 | 0.01863 | 10.49 | *osmY* | Osmotically-inducible protein Y |
| P0AGE9 | 0.00056 | 11.80 | *sucD* | Succinate--CoA ligase [ADP-forming] subunit alpha |
| P0A9G6 | 0.00578 | 22.74 | *aceA* | Isocitrate lyase |
| P0ABT2 | 0.01073 | 27.92 | *dps* | DNA protection during starvation protein |
