# Supplementary material for: Proteomic Study of the Survival and Resuscitation Mechanisms of Filamentous Persisters in an Evolved Escherichia coli Population from Cyclic Ampicillin Treatment
Source: mSystems. 2020 Jul 28;5(4):e00462-20. doi: 10.1128/mSystems.00462-20 (PMC7394356; doi:10.1128/mSystems.00462-20)
Supplement: TABLE S4 [file mSystems.00462-20-st004.docx]

| **UniProt ID** | **Gene** | **Protein name** | **Average spectral counts** |
| --- | --- | --- | --- |
| P0AFM6 | *pspA* | Phage shock protein A | 37.00 |
| P0AEE5 | *mglB* | D-galactose-binding periplasmic protein | 35.00 |
| P08997 | *aceB* | Malate synthase A | 34.33 |
| P0A836 | *sucC* | Succinate--CoA ligase [ADP-forming] subunit beta | 22.33 |
| P23538 | *ppsA* | Phosphoenolpyruvate synthase | 21.83 |
| P0AC38 | *aspA* | Aspartate ammonia-lyase | 21.33 |
| P09546 | *putA* | Bifunctional protein PutA | 20.00 |
| P23847 | *dppA* | Periplasmic dipeptide transport protein | 18.50 |
| P23843 | *oppA* | Periplasmic oligopeptide-binding protein | 17.17 |
| P0AET2 | *hdeB* | Acid stress chaperone HdeB | 17.00 |
| Q1PI72 | *icd* | Isocitrate dehydrogenase | 16.67 |
| P76558 | *maeB* | NADP-dependent malic enzyme | 16.50 |
| P0AC33 | *fumA* | Fumarate hydratase class I, aerobic | 15.33 |
| P0AB77 | *kbl* | 2-amino-3-ketobutyrate coenzyme A ligase | 14.50 |
| P07014 | *sdhB* | Succinate dehydrogenase iron-sulfur subunit | 14.33 |
| P0AG44 | *rplQ* | 50S ribosomal protein L17 | 13.50 |
| P00561 | *thrA* | Bifunctional aspartokinase/homoserine dehydrogenase 1 | 13.33 |
| P33599 | *nuoC* | NADH-quinone oxidoreductase subunit C/D | 13.17 |
| P0AAI3 | *ftsH* | ATP-dependent zinc metalloprotease FtsH | 12.67 |
| P0AEQ3 | *glnH* | Glutamine-binding periplasmic protein | 12.67 |
| P08194 | *glpT* | Glycerol-3-phosphate transporter | 12.50 |
| P0A9C0 | *glpA* | Anaerobic glycerol-3-phosphate dehydrogenase subunit A | 12.17 |
| P0ADE8 | *ygfZ* | tRNA-modifying protein YgfZ | 12.17 |
| P02930 | *tolC* | Outer membrane protein TolC | 12.00 |
| P0A991 | *fbaB* | Fructose-bisphosphate aldolase class 1 | 11.83 |
| P0A8G6 | *wrbA* | NAD(P)H dehydrogenase | 11.67 |
| P19926 | *agp* | Glucose-1-phosphatase | 11.50 |
| P00363 | *frdA* | Fumarate reductase flavoprotein subunit | 11.33 |
| P0ABI8 | *cyoB* | Cytochrome bo(3) ubiquinol oxidase subunit 1 | 11.33 |
| P0ABJ1 | *cyoA* | Cytochrome bo(3) ubiquinol oxidase subunit 2 | 11.00 |
| P0ADW3 | *yhcB* | Inner membrane protein YhcB | 10.67 |
| P27550 | *acs* | Acetyl-coenzyme A synthetase | 10.50 |
| P37095 | *pepB* | Peptidase B | 10.33 |
| P63224 | *gmhA* | Phosphoheptose isomerase | 9.83 |
| P00490 | *malP* | Maltodextrin phosphorylase | 9.00 |
| P75694 | *yahO* | Uncharacterized protein YahO | 8.67 |
| P0AAG8 | *mglA* | Galactose/methyl galactoside import ATP-binding protein MglA | 8.50 |
| P76116 | *yncE* | Uncharacterized protein YncE | 8.50 |
| P37330 | *glcB* | Malate synthase G | 8.33 |
| P0ACJ8 | *crp* | cAMP-activated global transcriptional regulator CRP | 8.17 |
| P45523 | *fkpA* | FKBP-type peptidyl-prolyl cis-trans isomerase FkpA | 8.00 |
| P0AD33 | *yfcZ* | UPF0381 protein YfcZ | 7.83 |
| P68206 | *yjbJ* | UPF0337 protein YjbJ | 7.83 |
| P0AE06 | *acrA* | Multidrug efflux pump subunit AcrA | 7.83 |
| P09372 | *grpE* | Protein GrpE | 7.83 |
| P27248 | *gcvT* | Aminomethyltransferase | 7.83 |
| P0AFK9 | *potD* | Spermidine/putrescine-binding periplasmic protein | 7.67 |
| P62768 | *yaeH* | UPF0325 protein YaeH | 7.67 |
| P0A6X7 | *ihfA* | Integration host factor subunit alpha | 7.50 |
| P0AFC7 | *nuoB* | NADH-quinone oxidoreductase subunit B | 7.33 |
| P0ADB7 | *ecnB* | Entericidin B | 7.17 |
| P25516 | *acnA* | Aconitate hydratase A | 7.00 |
| P0AEU0 | *hisJ* | Histidine-binding periplasmic protein | 6.83 |
| P0AC02 | *bamD* | Outer membrane protein assembly factor BamD | 6.67 |
| P76621 | *glaH* | Glutarate 2-hydroxylase | 6.67 |
| P07024 | *ushA* | Protein UshA [Includes: UDP-sugar hydrolase | 6.50 |
| P0ADA3 | *nlpD* | Murein hydrolase activator NlpD | 6.50 |
| P0AC62 | *grxC* | Glutaredoxin 3 | 6.50 |
| P0AG90 | *secD* | Protein translocase subunit SecD | 6.33 |
| P13445 | *rpoS* | RNA polymerase sigma factor RpoS | 6.33 |
| P0AC47 | *frdB* | Fumarate reductase iron-sulfur subunit | 6.00 |
| P26646 | *acuI* | Probable acrylyl-CoA reductase AcuI | 6.00 |
| P0A9A9 | *fur* | Ferric uptake regulation protein | 6.00 |
| P39180 | *flu* | Antigen 43 | 6.00 |
| P06715 | *gor* | Glutathione reductase | 5.83 |
| P0A9P4 | *trxB* | Thioredoxin reductase | 5.83 |
| P22256 | *gabT* | 4-aminobutyrate aminotransferase GabT | 5.67 |
| P00963 | *asnA* | Aspartate--ammonia ligase | 5.67 |
| P21179 | *katE* | Catalase HPII | 5.67 |
| P0A912 | *pal* | Peptidoglycan-associated lipoprotein | 5.67 |
| P0ABH9 | *clpA* | ATP-dependent Clp protease ATP-binding subunit ClpA | 5.50 |
| P52643 | *ldhA* | D-lactate dehydrogenase | 5.50 |
| P36938 | *pgm* | Phosphoglucomutase | 5.50 |
| P63020 | *nfuA* | Fe/S biogenesis protein NfuA | 5.33 |
| P0AGD3 | *sodB* | Superoxide dismutase | 5.33 |
| P0ACB7 | *hemY* | Protein HemY | 5.17 |
| P31658 | *hchA* | Protein/nucleic acid deglycase 1 | 5.17 |
