# Supplementary material for: Proteomic Study of the Survival and Resuscitation Mechanisms of Filamentous Persisters in an Evolved Escherichia coli Population from Cyclic Ampicillin Treatment
Source: mSystems. 2020 Jul 28;5(4):e00462-20. doi: 10.1128/mSystems.00462-20 (PMC7394356; doi:10.1128/mSystems.00462-20)
Supplement: TABLE S5 [file mSystems.00462-20-st005.docx]

| **Duration** | **UniProt ID** | **p-value** | **Folds** | **Gene** | **Protein name** |
| --- | --- | --- | --- | --- | --- |
| 1 h | P75691 | 0.00002 | 0.13 | *yahK* | Aldehyde reductase YahK |
|  | P0AC62 | 0.00385 | 0.22 | *grxC* | Glutaredoxin 3 |
|  | P27248 | 0.01849 | 0.23 | *gcvT* | Aminomethyltransferase |
|  | P37330 | 0.00704 | 0.27 | *glcB* | Malate synthase G |
|  | P0ABQ2 | 0.01588 | 0.31 | *garR* | 2-hydroxy-3-oxopropionate reductase |
|  | P31658 | 0.01314 | 0.41 | *hchA* | Protein/nucleic acid deglycase 1 |
|  | P27298 | 0.04397 | 0.41 | *prlC* | Oligopeptidase A |
|  | P21367 | 0.02834 | 0.43 | *ycaC* | Probable hydrolase YcaC |
|  | P0AFH8 | 0.02048 | 0.44 | *osmY* | Osmotically-inducible protein Y |
|  | P25516 | 0.00325 | 0.45 | *acnA* | Aconitate hydratase A |
|  | P18843 | 0.03298 | 0.49 | *nadE* | NH(3)-dependent NAD(+) synthetase |
|  | P68206 | 0.01745 | 0.49 | *yjbJ* | UPF0337 protein YjbJ |
|  | P23847 | 0.03039 | 0.50 | *dppA* | Periplasmic dipeptide transport protein |
|  | P0A9P4 | 0.01762 | 0.51 | *trxB* | Thioredoxin reductase |
|  | P21179 | 0.01086 | 0.56 | *katE* | Catalase HPII |
|  | P08200 | 0.04178 | 0.60 | *icd* | Isocitrate dehydrogenase |
|  | P0AAG8 | 0.03000 | 0.64 | *mglA* | Galactose/methyl galactoside import ATP-binding protein |
|  | P76558 | 0.02987 | 0.66 | *maeB* | NADP-dependent malic enzyme |
|  | P08312 | 0.03016 | 1.50 | *pheS* | Phenylalanine--tRNA ligase alpha subunit |
|  | P0AD61 | 0.00297 | 1.66 | *pykF* | Pyruvate kinase I |
|  | P0A7L0 | 0.04276 | 1.67 | *rplA* | 50S ribosomal protein L1 |
|  | P62399 | 0.04022 | 1.67 | *rplE* | 50S ribosomal protein L5 |
|  | P02413 | 0.04542 | 1.67 | *rplO* | 50S ribosomal protein L15 |
|  | P0A7V0 | 0.01100 | 1.80 | *rpsB* | 30S ribosomal protein S2 |
|  | P0A6Q6 | 0.00782 | 1.82 | *fabZ* | 3-hydroxyacyl-[acyl-carrier-protein] dehydratase FabZ |
|  | P28904 | 0.04175 | 2.00 | *treC* | Trehalose-6-phosphate hydrolase |
|  | P60422 | 0.04695 | 2.22 | *rplB* | 50S ribosomal protein L2 |
|  | P69811 | 0.03070 | 2.54 | *fruB* | Multiphosphoryl transfer protein |
|  | P0C018 | 0.04234 | 2.87 | *rplR* | 50S ribosomal protein L18 |
|  | P0A7R9 | 0.03469 | 2.97 | *rpsK* | 30S ribosomal protein S11 |
|  | P0ACE0 | 0.03424 | 3.84 | *hybC* | Hydrogenase-2 large chain |
|  | P0AEI1 | 0.04280 | 3.97 | *miaB* | tRNA-2-methylthio-N(6)-dimethylallyladenosine synthase |
|  | P0A9H3 | 0.01040 | 4.79 | *cadA* | Inducible lysine decarboxylase |
| 2 h | P21362 | 0.0095 | 0.16 | *yciF* | Protein YciF |
|  | P37330 | 0.0027 | 0.17 | *glcB* | Malate synthase G |
|  | P0AAV6 | 0.0498 | 0.28 | *ybgS* | Uncharacterized protein YbgS |
|  | P0A867 | 0.0498 | 0.29 | *talA* | Transaldolase A |
|  | P33570 | 0.0213 | 0.31 | *tktB* | Transketolase 2 |
|  | P75691 | 0.0467 | 0.34 | *yahK* | Aldehyde reductase YahK |
|  | P0AFH8 | 0.0046 | 0.35 | *osmY* | Osmotically-inducible protein Y |
|  | P25516 | 0.0412 | 0.35 | *acnA* | Aconitate hydratase A |
|  | P31658 | 0.0151 | 0.36 | *hchA* | Protein/nucleic acid deglycase 1 |
|  | P0AC62 | 0.0082 | 0.39 | *grxC* | Glutaredoxin 3 |
|  | P21179 | 0.0003 | 0.40 | *katE* | Catalase HPII |
|  | P18843 | 0.0103 | 0.46 | *nadE* | NH(3)-dependent NAD(+) synthetase |
|  | P68206 | 0.0324 | 0.49 | *yjbJ* | UPF0337 protein YjbJ |
|  | P0ADA3 | 0.0345 | 0.53 | *nlpD* | Murein hydrolase activator NlpD |
|  | P76658 | 0.0490 | 0.53 | *hldE* | Bifunctional protein HldE |
|  | P69776 | 0.0132 | 0.56 | *lpp* | Major outer membrane prolipoprotein Lpp |
|  | P08622 | 0.0215 | 0.56 | *dnaJ* | Chaperone protein DnaJ |
|  | P0A991 | 0.0448 | 0.58 | *fbaB* | Fructose-bisphosphate aldolase class 1 |
|  | P08997 | 0.0399 | 0.58 | *aceB* | Malate synthase A |
|  | P06715 | 0.0277 | 0.59 | *gor* | Glutathione reductase |
|  | P0AC38 | 0.0303 | 1.59 | *aspA* | Aspartate ammonia-lyase |
|  | P0A8F0 | 0.0450 | 1.63 | *upp* | Uracil phosphoribosyltransferase |
|  | P0ADY3 | 0.0309 | 1.66 | *rplN* | 50S ribosomal protein L14 |
|  | P0A6A3 | 0.0317 | 2.00 | *ackA* | Acetate kinase |
|  | P11875 | 0.0429 | 2.12 | *argS* | Arginine--tRNA ligase |
|  | P61316 | 0.0216 | 2.14 | *lolA* | Outer-membrane lipoprotein carrier protein |
|  | P62399 | 0.0103 | 2.18 | *rplE* | 50S ribosomal protein L5 |
|  | P69797 | 0.0034 | 2.35 | *manX* | PTS system mannose-specific EIIAB component |
|  | P0AE52 | 0.0029 | 2.53 | *bcp* | Peroxiredoxin Bcp |
|  | P68066 | 0.0030 | 2.58 | *grcA* | Autonomous glycyl radical cofactor |
|  | P0A7R9 | 0.0176 | 2.88 | *rpsK* | 30S ribosomal protein S11 |
|  | P0A707 | 0.0167 | 3.01 | *infC* | Translation initiation factor IF-3 |
|  | P76576 | 0.0331 | 3.05 | *yfgM* | UPF0070 protein YfgM |
|  | P0AEI1 | 0.0231 | 3.11 | *miaB* | tRNA-2-methylthio-N(6)-dimethylallyladenosine synthase |
|  | P0A9H3 | 0.0223 | 3.76 | *cadA* | Inducible lysine decarboxylase |
|  | P0ACE0 | 0.0269 | 5.04 | *hybC* | Hydrogenase-2 large chain |
| 3 h | P0A867 | 0.0321 | 0.27 | *talA* | Transaldolase A |
|  | P77747 | 0.0484 | 0.31 | *ompN* | Outer membrane porin N |
|  | P0A715 | 0.0206 | 0.32 | *kdsA* | 2-dehydro-3-deoxyphosphooctonate aldolase |
|  | P0AAG8 | 0.0317 | 0.38 | *mglA* | Galactose/methyl galactoside import ATP-binding protein |
|  | P0AFH8 | 0.0116 | 0.40 | *osmY* | Osmotically-inducible protein Y |
|  | P25516 | 0.0352 | 0.41 | *acnA* | Aconitate hydratase A |
|  | P21179 | 0.0003 | 0.41 | *katE* | Catalase HPII |
|  | P29131 | 0.0156 | 0.43 | *ftsN* | Cell division protein FtsN |
|  | P69783 | 0.0420 | 0.44 | *crr* | PTS system glucose-specific EIIA component |
|  | P08622 | 0.0073 | 0.47 | *dnaJ* | Chaperone protein DnaJ |
|  | P0ADE6 | 0.0050 | 0.47 | *kbp* | Potassium binding protein Kbp |
|  | P0A8E7 | 0.0326 | 0.48 | *yajQ* | UPF0234 protein YajQ |
|  | P69776 | 0.0163 | 0.48 | *lpp* | Major outer membrane prolipoprotein Lpp |
|  | P76658 | 0.0286 | 0.49 | *hldE* | Bifunctional protein HldE |
|  | P31658 | 0.0165 | 0.49 | *hchA* | Protein/nucleic acid deglycase 1 |
|  | P0ADA3 | 0.0247 | 0.50 | *nlpD* | Murein hydrolase activator NlpD |
|  | P0A7Z0 | 0.0152 | 0.50 | *rpiA* | Ribose-5-phosphate isomerase A |
|  | P68206 | 0.0226 | 0.52 | *yjbJ* | UPF0337 protein YjbJ |
|  | P18843 | 0.0425 | 0.60 | *nadE* | NH(3)-dependent NAD(+) synthetase |
|  | P0AB38 | 0.0224 | 0.61 | *lpoB* | Penicillin-binding protein activator LpoB |
|  | P0A9Q1 | 0.0161 | 0.61 | *arcA* | Aerobic respiration control protein ArcA |
|  | P0A8G6 | 0.0352 | 0.64 | *wrbA* | NAD(P)H dehydrogenase |
|  | P0A6A8 | 0.0152 | 1.51 | *acpP* | Acyl carrier protein |
|  | P21889 | 0.0096 | 1.54 | *aspS* | Aspartate--tRNA ligase |
|  | P0A7V0 | 0.0467 | 1.59 | *rpsB* | 30S ribosomal protein S2 |
|  | P0A717 | 0.0169 | 1.59 | *prs* | Ribose-phosphate pyrophosphokinase |
|  | P0A9P0 | 0.0007 | 1.62 | *lpdA* | Dihydrolipoyl dehydrogenase |
|  | P0AC38 | 0.0026 | 1.66 | *aspA* | Aspartate ammonia-lyase |
|  | P23843 | 0.0233 | 1.68 | *oppA* | Periplasmic oligopeptide-binding protein |
|  | P0AEE5 | 0.0061 | 1.68 | *mglB* | D-galactose-binding periplasmic protein |
|  | P33599 | 0.0396 | 1.69 | *nuoC* | NADH-quinone oxidoreductase subunit C/D |
|  | P08312 | 0.0298 | 1.74 | *pheS* | Phenylalanine--tRNA ligase alpha subunit |
|  | P0A7K6 | 0.0453 | 1.78 | *rplS* | 50S ribosomal protein L19 |
|  | P0A836 | 0.0038 | 1.83 | *sucC* | Succinate--CoA ligase [ADP-forming] subunit beta |
|  | P0A6B7 | 0.0306 | 1.85 | *iscS* | Cysteine desulfurase IscS |
|  | P07395 | 0.0132 | 1.89 | *pheT* | Phenylalanine--tRNA ligase beta subunit |
|  | P00934 | 0.0092 | 1.91 | *thrC* | Threonine synthase |
|  | P0A998 | 0.0149 | 2.04 | *ftnA* | Bacterial non-heme ferritin |
|  | P62399 | 0.0064 | 2.15 | *rplE* | 50S ribosomal protein L5 |
|  | P0A9X4 | 0.0024 | 2.26 | *mreB* | Cell shape-determining protein MreB |
|  | P13035 | 0.0037 | 2.27 | *glpD* | Aerobic glycerol-3-phosphate dehydrogenase |
|  | P13029 | 0.0473 | 2.36 | *katG* | Catalase-peroxidase |
|  | P07012 | 0.0397 | 2.44 | *prfB* | Peptide chain release factor RF2 |
|  | P61316 | 0.0101 | 2.48 | *lolA* | Outer-membrane lipoprotein carrier protein |
|  | P37902 | 0.0306 | 2.70 | *gltI* | Glutamate/aspartate import solute-binding protein |
|  | P60422 | 0.0168 | 2.72 | *rplB* | 50S ribosomal protein L2 |
|  | P00452 | 0.0187 | 3.10 | *nrdA* | Ribonucleoside-diphosphate reductase 1 subunit alpha |
|  | P77581 | 0.0261 | 3.19 | *astC* | Succinylornithine transaminase |
|  | P0A7R9 | 0.0022 | 3.20 | *rpsK* | 30S ribosomal protein S11 |
|  | P13445 | 0.0361 | 3.21 | *rpoS* | RNA polymerase sigma factor RpoS |
|  | P0AEI1 | 0.0349 | 3.30 | *miaB* | tRNA-2-methylthio-N(6)-dimethylallyladenosine synthase |
|  | P0AE52 | 0.0095 | 3.31 | *bcp* | Peroxiredoxin Bcp |
|  | P0A7T3 | 0.0411 | 3.31 | *rpsP* | 30S ribosomal protein S16 |
|  | P37330 | 0.0015 | 3.32 | *glcB* | Malate synthase G |
|  | P27550 | 0.0005 | 3.50 | *acs* | Acetyl-coenzyme A synthetase |
|  | P15288 | 0.0228 | 3.50 | *pepD* | Cytosol non-specific dipeptidase |
|  | P0AFD6 | 0.0202 | 3.54 | *nuoI* | NADH-quinone oxidoreductase subunit I |
|  | P33195 | 0.0317 | 3.59 | *gcvP* | Glycine dehydrogenase |
|  | P0C8J8 | 0.0431 | 3.75 | *gatZ* | D-tagatose-1,6-bisphosphate aldolase subunit GatZ |
|  | P03841 | 0.0098 | 3.85 | *malM* | Maltose operon periplasmic protein |
|  | P0ACE0 | 0.0335 | 3.94 | *hybC* | Hydrogenase-2 large chain |
|  | P21420 | 0.0208 | 3.95 | *nmpC* | Putative outer membrane porin protein NmpC |
|  | P23882 | 0.0056 | 4.03 | *fmt* | Methionyl-tRNA formyltransferase |
|  | P0A707 | 0.0303 | 4.39 | *infC* | Translation initiation factor IF-3 |
|  | P09147 | 0.0471 | 4.54 | *galE* | UDP-glucose 4-epimerase |
|  | P00582 | 0.0395 | 4.77 | *polA* | DNA polymerase I |
|  | P0A763 | 0.0043 | 5.90 | *ndk* | Nucleoside diphosphate kinase |
|  | P0AG86 | 0.0175 | 5.90 | *secB* | Protein-export protein SecB |
|  | P08331 | 0.0321 | 6.08 | *cpdB* | 2',3'-cyclic-nucleotide 2'-phosphodiesterase/3'-nucleotidase |
|  | P0A8E1 | 0.0014 | 7.26 | *ycfP* | UPF0227 protein YcfP |
|  | P0A9K9 | 0.0281 | 10.29 | *slyD* | FKBP-type peptidyl-prolyl cis-trans isomerase SlyD |
