# Supplementary material for: Proteomic Study of the Survival and Resuscitation Mechanisms of Filamentous Persisters in an Evolved Escherichia coli Population from Cyclic Ampicillin Treatment
Source: mSystems. 2020 Jul 28;5(4):e00462-20. doi: 10.1128/mSystems.00462-20 (PMC7394356; doi:10.1128/mSystems.00462-20)
Supplement: TABLE S6 [file mSystems.00462-20-st006.docx]

| **UniProt ID** | **p-value** | **Folds** | **Gene** | **Protein name** |
| --- | --- | --- | --- | --- |
| P25553 | 0.00174 | 0.13 | *aldA* | Lactaldehyde dehydrogenase |
| P0ABH7 | 0.00136 | 0.16 | *gltA* | Citrate synthase |
| P0AFH8 | 0.00858 | 0.19 | *osmY* | Osmotically-inducible protein Y |
| P0A799 | 0.01104 | 0.24 | *pgk* | Phosphoglycerate kinase |
| P0ADZ7 | 0.01504 | 0.26 | *yajC* | Sec translocon accessory complex subunit YajC |
| P00956 | 0.00017 | 0.27 | *ileS* | Isoleucine--tRNA ligase |
| P0AE08 | 0.01629 | 0.28 | *ahpC* | Alkyl hydroperoxide reductase C |
| P0A9G6 | 0.00260 | 0.30 | *aceA* | Isocitrate lyase |
| P0AFF6 | 0.00467 | 0.31 | *nusA* | Transcription termination/antitermination protein NusA |
| P08997 | 0.00549 | 0.32 | *aceB* | Malate synthase A |
| P09373 | 0.01248 | 0.34 | *pflB* | Formate acetyltransferase 1 |
| P09546 | 0.00719 | 0.36 | *putA* | Bifunctional protein PutA |
| P0ABB4 | 0.01108 | 0.49 | *atpD* | ATP synthase subunit beta |
| P0A853 | 0.00343 | 0.49 | *tnaA* | Tryptophanase |
| P0ABB0 | 0.00679 | 0.63 | *atpA* | ATP synthase subunit alpha |
| P06996 | 0.00134 | 2.05 | *ompC* | Outer membrane porin C |
| P02931 | 0.00364 | 2.23 | *ompF* | Outer membrane porin F |
| P02359 | 0.00901 | 2.35 | *rpsG* | 30S ribosomal protein S7 |
| P0A8T7 | 0.01573 | 2.41 | *rpoC* | DNA-directed RNA polymerase subunit beta' |
| P0AG99 | 0.01396 | 2.69 | *secG* | Protein-export membrane protein SecG |
| P06959 | 0.01413 | 2.80 | *aceF* | Dihydrolipoyllysine-residue acetyltransferase component of pyruvate dehydrogenase complex |
| P0A6P1 | 0.01462 | 2.86 | *tsf* | Elongation factor Ts |
| P0AG55 | 0.01229 | 3.05 | *rplF* | 50S ribosomal protein L6 |
| P77747 | 0.01243 | 3.59 | *ompN* | Outer membrane porin N |
| P02358 | 0.01033 | 4.30 | *rpsF* | 30S ribosomal protein S6 |
| P60624 | 0.00016 | 4.39 | *rplX* | 50S ribosomal protein L24 |
| P0AEH5 | 0.00015 | 7.27 | *elaB* | Protein ElaB |
| P0ADZ0 | 0.00861 | 10.20 | *rplW* | 50S ribosomal protein L23 |
