# Supplementary material for: Proteomic Study of the Survival and Resuscitation Mechanisms of Filamentous Persisters in an Evolved Escherichia coli Population from Cyclic Ampicillin Treatment
Source: mSystems. 2020 Jul 28;5(4):e00462-20. doi: 10.1128/mSystems.00462-20 (PMC7394356; doi:10.1128/mSystems.00462-20)
Supplement: TABLE S7 [file mSystems.00462-20-st007.docx]

| **Gene** | **Protein name** | **Protein expression** |
| --- | --- | --- |
| *atpA* | ATP synthase subunit alpha | Increasing expression profile from 1h to 5h of treatment |
| *atpD* | ATP synthase subunit beta | Up-regulated after 5h of treatment |
| *atpG* | ATP synthase gamma chain | Up-regulated after 5h of treatment |
| *nuoB* | NADH-quinone oxidoreductase subunit B | Newly detected on 5h of treatment |
| *nuoC* | NADH-quinone oxidoreductase subunit C/D | Newly detected on 5h of treatment |
| *cyoA* | Cytochrome bo(3) ubiquinol oxidase subunit 2 | Newly detected on 5h of treatment |
| *cyoB* | Cytochrome bo(3) ubiquinol oxidase subunit 1 | Newly detected on 5h of treatment |
| *sdhA* | Succinate dehydrogenase flavoprotein subunit | Up-regulated after 5h of treatment |
| *sdhB* | Succinate dehydrogenase iron-sulfur subunit | Newly detected on 5h of treatment |
| *sucC* | Succinate—CoA ligase [ADP-forming] subunit beta | Newly detected on 5h of treatment |
| *sucD* | Succinate—CoA ligase [ADP-forming] subunit alpha | Up-regulated after 5h of treatment |
| *pta* | Phosphate acetyltransferase | Down-regulated after 5h of treatment |
